# Superresolution microscopy reveals distinct localisation of full length IRSp53 and its I-BAR domain protein within filopodia

**DOI:** 10.1038/s41598-019-38851-w

**Published:** 2019-02-21

**Authors:** Thankiah Sudhaharan, Srivats Hariharan, John Soon Yew Lim, Jaron Zhongliang Liu, Yen Ling Koon, Graham D. Wright, Keng Hwee Chiam, Sohail Ahmed

**Affiliations:** 1 Institute of Medical Biology, A*STAR, Singapore, 138684 Singapore; 2 Skin Research Institute of Singapore, A*STAR, Singapore, 138648 Singapore; 3 Bioinformatics Institute, A*STAR, Singapore, 138671 Singapore; 4 Present Address: Olympus Singapore Pte Ltd., Singapore, 248373 Singapore; 5 Present Address: GE Healthcare, Singapore, 099253 Singapore

## Abstract

Superresolution microscopy offers the advantage of imaging biological structures within cells at the nano-scale. Here we apply two superresolution microscopy techniques, specifically 3D structured illumination microscopy (3D-SIM) and direct stochastic optical reconstruction microscopy (dSTORM), a type of single molecule localisation microscopy, to localise IRSp53 protein and its I-BAR domain in relation to F-actin within filopodia. IRSp53 generates dynamic (extending and retracting) filopodia 300 nm wide with a distinct gap between IRSp53 and F-actin. By contrast, protrusions induced by the I-BAR domain alone are non-dynamic measuring between 100–200 nm in width and exhibit a comparatively closer localisation of the I-BAR domain with the F-actin. The data suggest that IRSp53 membrane localisation is spatially segregated to the lateral edges of filopodia, in contrast to the I-BAR domain is uniformly distributed throughout the membranes of protrusions. Modeling of fluorescence recovery after photobleaching (FRAP) data suggests that a greater proportion of I-BAR domain is associated with membranes when compared to full length IRSp53. The significance of this new data relates to the role filopodia play in cell migration and its importance to cancer.

## Introduction

The development of superresolution microscopy, enabling biologists to surpass the diffraction limit of light microscopy, has been a revolutionary tool as we strive to understand the biology of cells. Superresolution microscopy, when combined with the specificity of fluorescence labelling techniques, makes it possible to image cellular structures with nano-scale resolution. Among the various superresolution approaches available, 3D structured illumination microscopy^1,2^ (3D-SIM) offers resolution in the range of 110–130 nm, whereas single molecules localisation microscopy (SMLM) techniques such as photo-activated localisation microscopy^3^ (PALM), stochastic optical reconstruction microscopy^4^ (STORM) and direct STORM^5,6^ (dSTORM) push the resolution down around 20–30 nm. Whilst not applied in the current work, stimulated emission depletion (STED) microscopy is the third major category of superresolution techniques, offering an intermediate resolution between SMLM and 3D-SIM. Numerous examples in which superresolution microscopy has been applied to biological research are available in the literature; for example, Bates *et al*.^7^ have demonstrated the width of mammalian microtubules to be ~50 nm using multicolour SMLM with photo-switchable fluorescent probes and both Horn *et al*.^8^ and Schücker *et al*.^9^ have studied the proteins involved in the formation and organisation of the synaptonemal complex using 3D-SIM and dSTORM, respectively. Studies using time-resolved STORM on filopodia extension and retraction, including membrane dynamics using DiI labelling, has achieved a resolution of ~30–60 nm in living cells^10^. Hsu *et al*.^11^ characterized the dynamics of cancer cell filopodia, accomplishing a lateral resolution of ~120 nm in live lung cancer cells applying a more developmental bright-field superresolution microscopy technique. However no attempt has been made to understand protein localisation and filopodial architecture at superresolution nanoscales.

Filopodia are actin-based membrane protrusions common to all mammalian cells that facilitate changes in cell morphology and are associated with lamellipodia and membrane ruffles. Filopodia are involved in cell motility, axon guidance, cell-cell contact and embryo development^12^. The formation of filopodia is regulated through several proteins that work downstream of membrane receptors. Specifically Rho GTP binding proteins Cdc42^13^ and Rif (Rho in filopodia) work with I-BAR (Inverse bin-amphiphysin-Rvs) domain proteins, IRSp53 (Insulin receptor substrate protein 53 kDa) and IRTKS^14^ (Insulin receptor tyrosine kinase substrate, also known as BAIAP2L1 or BAI1-associated protein 2-like 1) to initiate filopodia formation. The I-BAR family of proteins, IRSp53, IRTKS, MIM (Missing in metastasis), ABBA (actin-bundling protein with BAIAP2 homology), and Pinkbar (Planar intestinal-and kidney-specific BAR domain protein, also known as BAI1-associated protein 2-like protein 2) combine membrane curvature related activity with actin dynamics and protein-protein interaction^15^.

Cdc42-activated IRSp53 localises to membranes, and generates peripheral filopodia working together with a variety of actin modulators (e.g. Mena^16^, Eps8^17^, N-WASP^18^, Dynamin^19^, mDia1^20^). By contrast, IRTKS works with Rif to generate dorsal filopodia in a pathway that utilizes Eps8 and WAVE2^14^. Pinkbar is another target of Rif involved in the generation of membrane sheets associated with cell-cell contact^21^. Taken together these studies show that Rho GTPases control aspects of cell morphology through the I-BAR family of proteins. *In vitro* experiments with Giant Unilamellar Vesicles (GUV) model membranes have demonstrated that the I-BAR domain can oligomerise and deform membranes to generate actin-free ‘*protrusions’* of a defined length and width in the absence of actin^22^ (*‘protrusions’* will be used throughout the current work to describe structures generated by the I-BAR domain, in contrast to ‘*filopodia’* that will be used to define structures generated by full length IRSp53). An interesting and relevant observation was that I-BAR domains from different full length protein produced protrusions of different length and width^22^.

In previous work, using dual channel time-lapse microscopy, we demonstrated the time dependent recruitment specific actin modulators Dynamin, Mena and Eps8, by IRSp53^19^ during a filopodia’s life-cycle. Dynamin concentrates during filopodia formation, followed by Mena during the elongation phase and finally Eps8, during the retraction phase. The data in the present work shows that IRSp53, IRTKS, MIM and ABBA are able to generate dynamic (extending and retracting) filopodia while the I-BAR domain of these proteins alone generate non-dynamic (stable) protrusions. Given the key role of filopodia in cell-migration and cancer^23^, understanding their molecular architecture and composition is of great importance. The present work concentrates on applying 3D-SIM and dSTORM superresolution microscopy techniques to characterise the IRSp53 and its I-BAR domain within filopodia, as selected representatives of I-BAR domain family proteins.

## Results

### I-BAR family proteins generation of F-actin associated membranes protrusions

**Figure 1:**
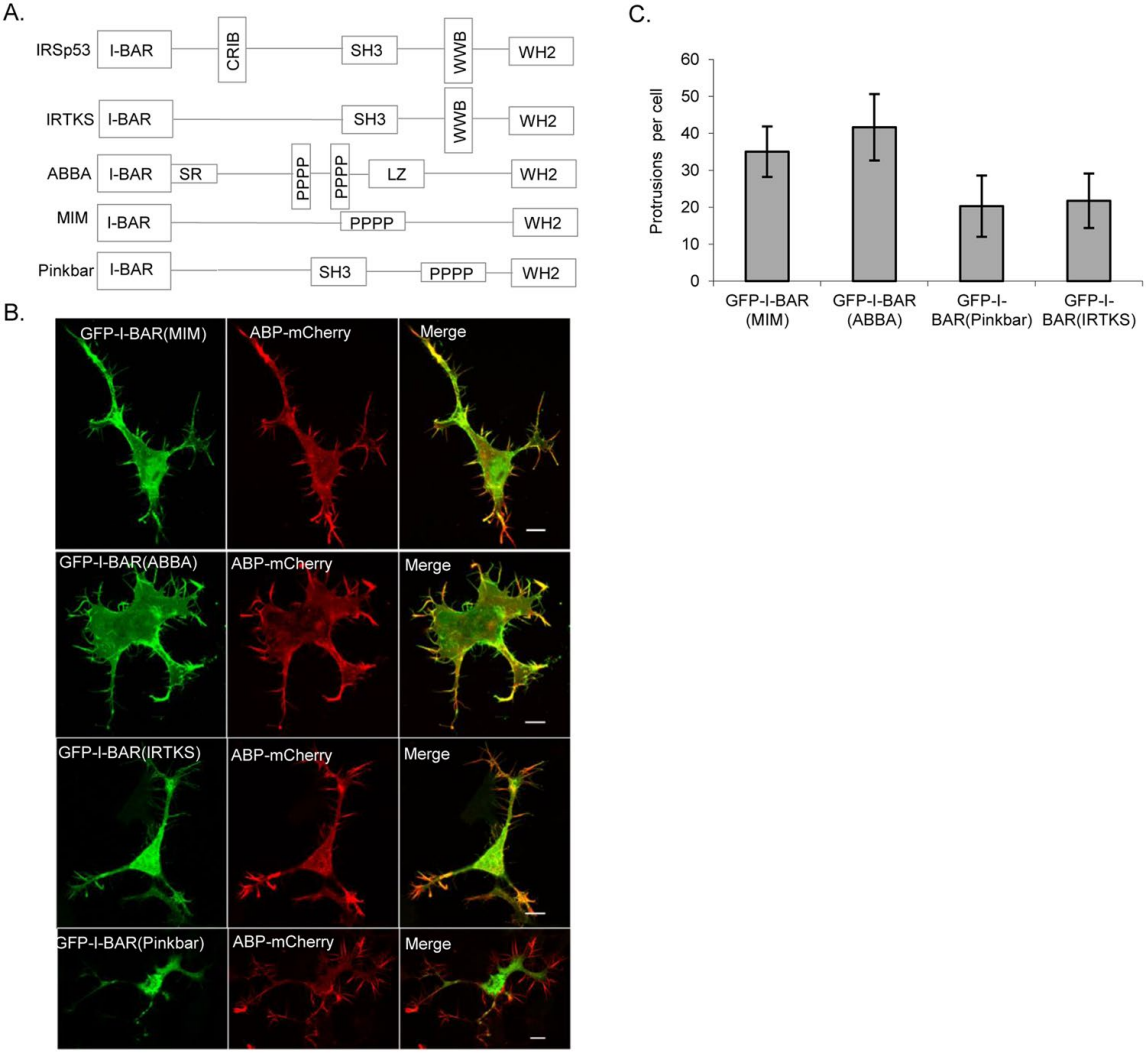
Filopodial characterization of I-BAR family proteins on N1E-115 cells. (**A**) Schematics of I-BAR family proteins domain structure. I-BAR domain, and C-terminus -WH2 domain are common among I-BAR family proteins. IRSp53, IRTKS and Pinkbar all have the -SH3 domain in common. The members of the family lack the CRIB domain with an exception of IRSp53. WW binding domain (WWB) is common among IRSp53 and IRTKS while the rest of the members are having proline rich region (PPPP) with the exception of ABBA having additional serine rich (SZ) and leucine zipper region (LZ). (**B**) Representative confocal images of N1E-115 cells co-expressing I-BAR domains of I-BAR family proteins fused with GFP and actin binding protein ABP-mCherry. Scale bar = 10 µm. (**C**) Average (mean) number of filopodia/protrusions generated per cell for cells such as those depicted in (**B**). Error bars show standard deviation (n = 7–12 cells).

To characterize the filopodia generating ability of I-BAR proteins, we first compared all members of the family. The full length IRSp53 and IRTKS members of the I-BAR family proteins have been shown to induce filopodia downstream of Cdc42 and Rif, respectively^13,14^. The domain schematics of the I-BAR family proteins^13^ (Fig. 1A). In-order to compare the phenotypic characteristic of full-length ABBA and MIM proteins with other I-BAR family proteins, we investigated whether each are able to induce filopodia. Mouse neuroblastoma N1E-115 cells were transfected with the relevant cDNAs including mCherry-ABP (actin binding protein). Wide-field fluorescence time-lapse microscopy was then used to follow cell morphology and filopodia dynamics in these cell lines (Fig. 1B). MIM and ABBA were both able to form filopodia with lifetimes similar to those observed when induced by IRSp53 and IRTKS^14,18^ (Table 1). Next, we investigated the morphological effects of I-BAR domains of MIM, ABBA, IRTKS, and Pinkbar using the well-characterised IRSp53 as a control. Our previous work^18^ has shown that the I-BAR domain of IRSp53 generates a variety of non-dynamic protrusions with lifetimes of >600 seconds in N-WASP WT cells, whereas IRSp53 filopodia exhibit lifetimes of 142 ± 101 seconds in N1E-115 cells. In this study cDNAs encoding GFP tagged I-BAR domains of MIM, ABBA, IRTKS and Pinkbar were co-expressed with mCherry-ABP in N1E-115 cells and membrane morphology imaged using live-cell and confocal microscopy. In all cases membrane protrusions were generated, but they were non-dynamic with lifetimes greater than 600 seconds (Fig. 1B,C and Table 2) in contrast to ~150–300 seconds for the full-length proteins (Table 1).

**Table 1:** I-BAR family proteins filopodial characterization in N1E-115 cells.

Protein(s) expressed	Filopodia per cell	Filopodial lifetime (s)	Filopodial length (µm)
GFP-actin*^14^	1.2 ± 1.0	212 ± 64	7.16 ± 0.82
mCherry-ABP*^20^	1.3 ± 1.0	166 ± 17	4.88 ± 1.01
GFP-MIM + mCherry-ABP	36.7 ± 5.6	281 ± 78	4.94 ± 1.06
GFP-ABBA + mCherry-ABP	35.3 ± 6.8	240 ± 98	5.23 ± 1.17
GFP-Pinkbar + mCherry-ABP	10.7 ± 2.6	163 ± 58	1.84 ± 0.27

Filopodial characterization was carried out on N1E-115 cells expressing respective full lengths I-BAR family proteins and mCherry-actin binding protein (ABP). Time-lapse microscopy was carried out 24 h post-transfection. The data represent the scoring for number of filopodia per cell, filopodial lifetime and filopodial length^18^ as previously reported. Data is expressed as mean, +/− s.d (n = 10 cells). *Data from previously reported results is shown for comparison but excluded from statistical analysis.

**Table 2:** I-BAR domain of I-BAR family proteins filopodial lifetime scoring in N1E-115 cells.

Protein(s) expressed	Protrusions lifetime (s)
I-BAR (MIM)	>600
GFP-I-BAR (ABBA)	>600
GFP-I-BAR (IRTKS)	>600
GFP-I-BAR (Pinkbar)	>600

Filopodial lifetime scoring was performed on N1E-115 cells expressing respective I-BAR domain of I-BAR family proteins and mCherry-actin binding protein (ABP). Time-lapse microscopy was carried out 24 h post-transfection (n = 7–12 cells).

### Single Molecule Localisation Microscopy of IRSp53 generated filopodia

To understand the spatial organisation of proteins within filopodia we applied single colour dSTORM to compare and contrast the cellular localisation of IRSp53 with its I-BAR domain. IRSp53 was selected as a model for all the I-BAR family of proteins as it is the best characterised. dSTORM has the ability to resolve cellular structures down to around 20 nm which compares to ~240 nm of typical confocal microscopy. Supplementary Fig. S1 shows the schematic of our home-built dSTORM system used in the current work. In order to test the experimental set up, we carried out a control experiment by labelling HA-IRSp53 expressed in N1E-115 cells with Alexa Fluor 647-conjugated anti-HA antibodies (Supplementary Fig. S2A, widefield fluorescence). The single molecule blinking (Supplementary Fig. S2B, and Movie S1) of the Alexa 647 was optimised in an imaging buffer of 100 mM MEA at pH 8.4.

**Figure 2:**
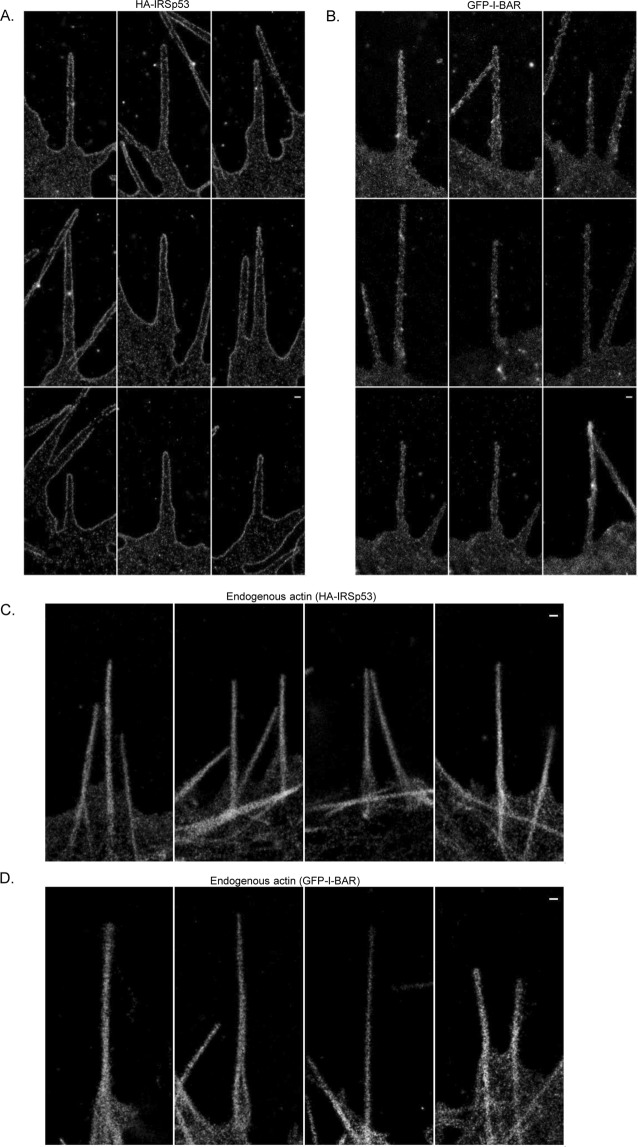
Single colour dSTORM images of N1E-115 cells expressing IRSp53, I-BAR and endogenous actin. (**A**) Filopodia expressing HA-IRSp53. Cells were labelled with anti-HA Alexa 647 conjugate for dSTORM imaging. (**B**) Filopodia expressing GFP-I-BAR (domain of IRSp53). Cells were labelled with anti-GFP Alexa 647 conjugate for dSTORM imaging. (**C**) Endogenous actin of HA-IRSp53 expressing cells. Cells were labelled with Alexa 647 Phalloidin. (**D**) Endogenous actin of GFP-I-BAR expressing cells. Cells were labelled with Alexa 647 Phalloidin. Scale bar = 200 nm throughout.

These optimised dSTORM imaging conditions were then used to acquire data for filopodia of N1E-115 cells expressing IRSp53, I-BAR domain of IRSp53 and endogenous actin. Raw dSTORM image data was processed for molecular localisations to generate the final superresolution images. Interestingly it was observed that IRSp53 is localised specifically in a spatially segregated pattern on the lateral edge of filopodia and not uniformly distributed throughout the membrane (Fig. 2A). The data revealed that the width of the filopodia of IRSp53 is around ~300 nm. By contrast the I-BAR domain of IRSp53 protein, expressed as GFP-fusion protein and labelled with anti-GFP Alexa 647 conjugate, was uniformly distributed throughout the protrusion (Fig. 2B). The data revealed that the width of the protrusion of I-BAR is around ~200 nm. dSTORM of endogenous actin labelled with Alexa 647 Phalloidin reveals that actin is localised in the middle of filopodia/protrusions as a single structure (~100 nm wide) for both full length IRSp53 (Fig. 2C) and I-BAR domain of IRSp53 (Fig. 2D). In addition, IRSp53 generates diverse membranes morphologies (Supplementary Fig. S2C) of filopodia in HeLa cells. Taken together, the single colour dSTORM data demonstrates that IRSp53, I-BAR domain of IRSp53 and actin proteins occupy different positions within filopodia.

To re-confirm the generated data, we repeated the dSTORM of full length IRSp53 protein by expressing GFP fusion protein of IRSp53 in Clontech pEGFP-C1 vector (Supplementary Fig. S3A(i)) and observed no variation in protein localisation within filopodia (*cf*. Fig. 2A). The other members of the I-BAR family proteins, IRTKS (Supplementary Fig. S3A(ii)), MIM (Supplementary Fig. S3A(iii)) and ABBA (Supplementary Fig. S3A(iv)), showed similar localisations. To investigate further the membrane association of IRSp53 localisation within filopodia, we repeated the dSTORM with cells expressing the membrane markers GFP-PH (pleckstrin homology domain of PLC) or PMT-YFP (plasma membrane targeted sequence fused with YFP) in a HA-IRSp53 background. The GFP-PH and PMT-YFP were labelled with anti-GFP-Alexa 647 (which also recognises YFP) to enable dSTORM. Both GFP-PH and PMT-YFP (Supplementary Fig. S3B), were localised throughout the filopodia, similar to the I-BAR domain localization (*cf*. Fig. 2B).

To confirm the plasma membrane localisation of I-BAR domain proteins, we utilised plasma membrane specific stains wheat germ agglutinin (WGA-594) and CellMask orange (CM-555). The plasma membrane specific localisation of PMT-YFP in N1E cell was verified with WGA-594 (Supplementary Fig. S4A). The plasma membrane localisation of GFP-I-BAR in filopodia was established with co-labelling with WGA-594 confocal microscopy, confirmed with a spatial intensity plot (Supplementary Fig. S4B,C). This was confirmed combining GFP-I-BAR and CM-555 dye in 3D-SIM imaging (Supplementary Fig. S4D). Taken together this data establishes that GFP-I-BAR domain protein is uniformly distributed at the plasma membrane of filopodia.

### Analysis of single colour dSTORM data

**Figure 3:**
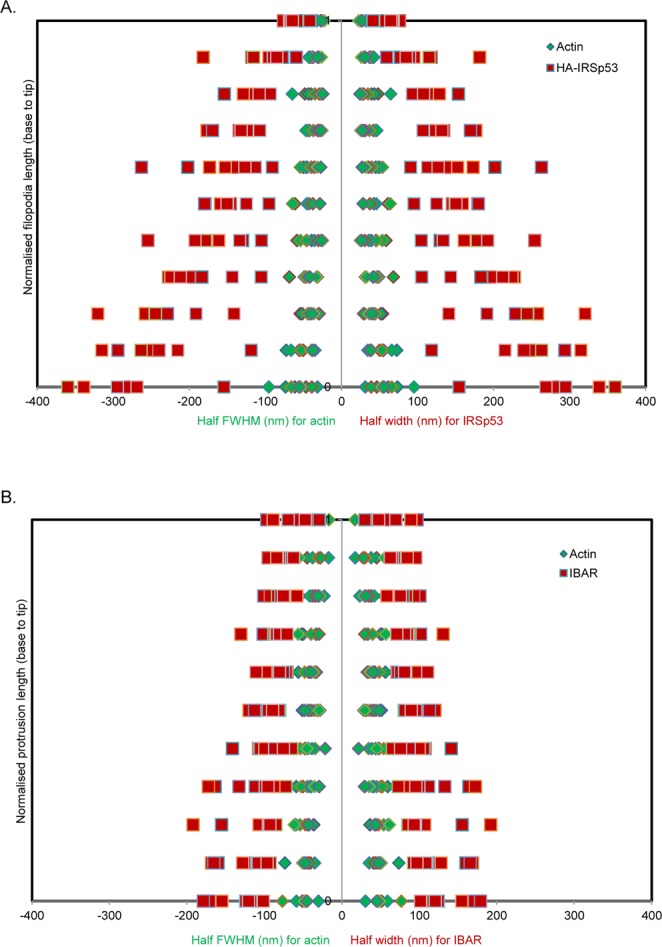
Width measurements of filopodia/protrusions/actin in N1E-115 cells from single colour dSTORM filopodia image. (**A**) Width measurement of filopodia on cells either expressing and labelled for HA-IRSp53 (red squares) or labelled for endogenous actin (green diamonds). For HA-IRSp53 data, either single or double Gaussian fitting was applied with subsequent FWHM measurements to enable width measurements (refer to Methods and Supplementary Fig. S2D for further details) (n = 12 for each label). (**B**) Width measurement of protrusion on cells either expressing GFP-I-BAR domain of IRSp53 (red squares) or endogenous actin (green diamonds). Either manual measurements or single Gaussian fitting with subsequent FWHM measurements enabled protrusion/actins widths to be determined (refer to Methods and Supplementary Fig. S2D for further details) (n = 12).

To quantitatively compare IRSp53/F-actin and I-BAR domain/F-actin localisations, the full width half maxima (FWHM) was calculated for each protein from single colour dSTORM data (approach described in Methods and depicted in Supplementary Fig. S2D). Figure 3A,B shows the plots derived demonstrating the relative positions. Figure 3A reveals that IRSp53 is localized at the sides of a central F-actin core structure with a width of ~300 nm (the F-actin was ~100 nm wide, within the filopodia). The ~150–200 nm space between IRSp53 and F-actin suggests that two proteins may not interact directly with each other except with a possibility in filopodial tips where they are more closely located. In contrast, Fig. 3B reveals the I-BAR domain formed protrusions of ~200 nm in width. The F-actin was localised in the core of the protrusion, with a width of ~100 nm. This result suggests a possibility of direct interaction between I-BAR and F-actin.

### Dual colour 3D-SIM and dSTORM imaging of filopodia

**Figure 4:**
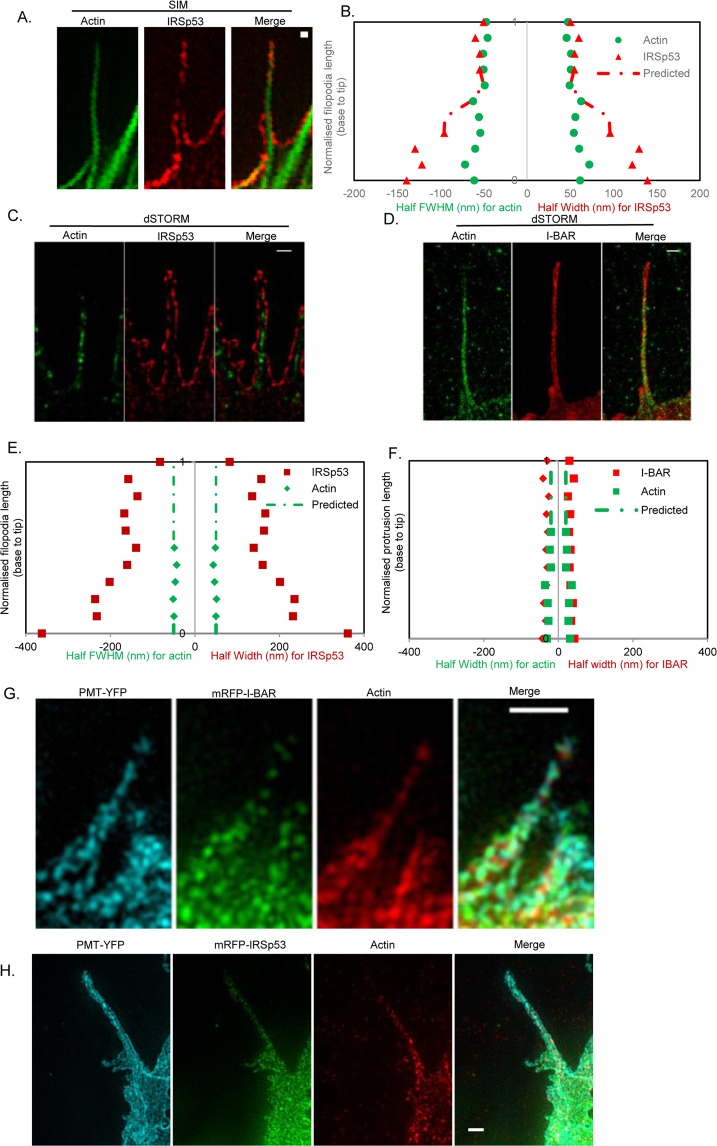
Representative dual colour dSTORM and 3D-SIM images of I-BAR/IRSp53-actin and three colour 3D-SIM images of PMT-YFP, I-BAR/IRSp53-actin in N1E-115 filopodia/protrusions. (**A**) Dual colour 3D-SIM of filopodia expressing HA-IRSp53 labelled for -HA using anti-HA Alexa 647 conjugate. Endogenous actin was labelled with Alexa 568 Phalloidin. Scale bar = 200 nm. (**B**) Width measurement (as previously described) was carried out for the filopodia shown in (**A**). Predicted values (interpolated) are used to represent the gaps in filopodia staining, based on the assumption that the filopodia will continue along the same path. (**C**) Dual colour dSTORM images of filopodia expressing HA-IRSp53 labelled with anti-HA Alexa 647 conjugate and endogenous actin with Alexa 568 Phalloidin. Scale bar = 200 nm. (**D**) Dual colour dSTORM images of filopodia expressing GFP-I-BAR domain of IRSp53 labelled with anti-GFP Alexa 647 conjugate and Alexa 568 Phalloidin. Scale bar = 200 nm. (**E**) Width measurement was carried out for the filopodia shown in (**C**). (**F**) Width measurement was carried out for the filopodia shown in (**D**). (**G**) Three colour 3D-SIM images of filopodia co-expressing PMT-YFP and mRFP-I-BAR. Cells were labelled as described in methods. Briefly, PMT-YFP (Alexa 488, cyan), mRFP-I-BAR (Alexa 568, green) and endogenous actin (Alexa 647 Phalloidin, red). Scale bar = 1 µm. (**H**) Three colour 3D-SIM image of filopodia co-expressing PMT-YFP and mRFP-IRSp53. Cells were labelled as described in methods. Briefly, PMT-YFP (Alexa 488, cyan), mRFP-IRSp53 (Alexa 568, green) and endogenous actin (Alexa 647 Phalloidin, red). Scale bar = 1 µm.

To take the observations from single colour dSTORM further we used dual colour 3D-SIM which can resolve between 110–130 nm. HA-IRSp53 expressing fixed N1E-115 cells were labelled using anti-HA Alexa 647 conjugate and endogenous F-actin was labelled with Alexa 568 Phalloidin and imaged with 3D-SIM. Despite the relatively lower resolution of 3D-SIM Fig. 4A,B confirms the quantitation presented in Fig. 3A, showing IRSp53 is spatially segregated to the lateral edge of filopodia and F-actin is localised to the central position with little contact except at the tips.

Dual colour dSTORM is a much more challenging experiment due to the need to make a compromise on the optimal condition for both dyes and consequent reduced blinking efficiency. However, given the dimensions of the filopodia and resolution limits of confocal and 3D-SIM, dual colour dSTORM is the most suitable technique to take the present analysis further. The blinking of both HA-IRSp53 with anti-HA-Alexa 647 and F-actin using Phalloidin Alexa 568 was optimised with an imaging buffer containing 100 mM MEA at pH 8.0. A typical region of interest showing both labels in an N1E-115 cell is shown in Supplementary Fig. S5; the widefield fluorescence region of interest for imaging (i & ii) and examples of blinking for both channels (iii & iv & Supplementary Movies S2A and S2B). A representative dSTORM image of filopodia/protrusion of IRSp53 and I-BAR domain of IRSp53 along with endogenous F-actin is shown in Fig. 4C,D together with their calculated FWHM in Fig. 4E,F, respectively. From these data, we can clearly see that there is no overlapping localisation between IRSp53 and F-actin. The compromise made when conducting dual colour dSTORM is evident through the non-continuous actin and membrane structures and poorer reconstructed images (*cf*. Fig. 2). These observations are in full agreements with the 3D-SIM data (Fig. 4A). By contrast, there is extensive overlap between the I-BAR domain of IRSp53 and F-actin. Additional dual colour dSTORM data is shown in Supplementary Fig. S4B,C.

To further verify the membrane and actin localisation differences between I-BAR domain and IRSp53, we performed three color 3D-SIM. A co-transfection of PMT-YFP and mRFP-I-BAR/IRSp53 was used as membrane specific dyes could not be used when a permeabilisation step is required. The fusion proteins were further labelled with respective primary antibodies and secondary antibodies, Alexa 488 for PMT-YFP and Alexa 568 secondary antibody for I-BAR/IRSp53 together with Alexa 647 Phalloidin for endogenous actin (Fig. 4G,H and Supplementary Movies S3 and S4, for I-BAR and IRSp53, respectively). Whilst the resolution is more limited when compared to dSTORM, both 3D and 3 colour imaging is possible with 3D-SIM. The data further supports the earlier 2D data and enables us to propose a model for filopodial proteins organisation.

### F-actin is required for filopodia formation by IRSp53 but not the protrusions generated by the I-BAR domain

**Figure 5:**
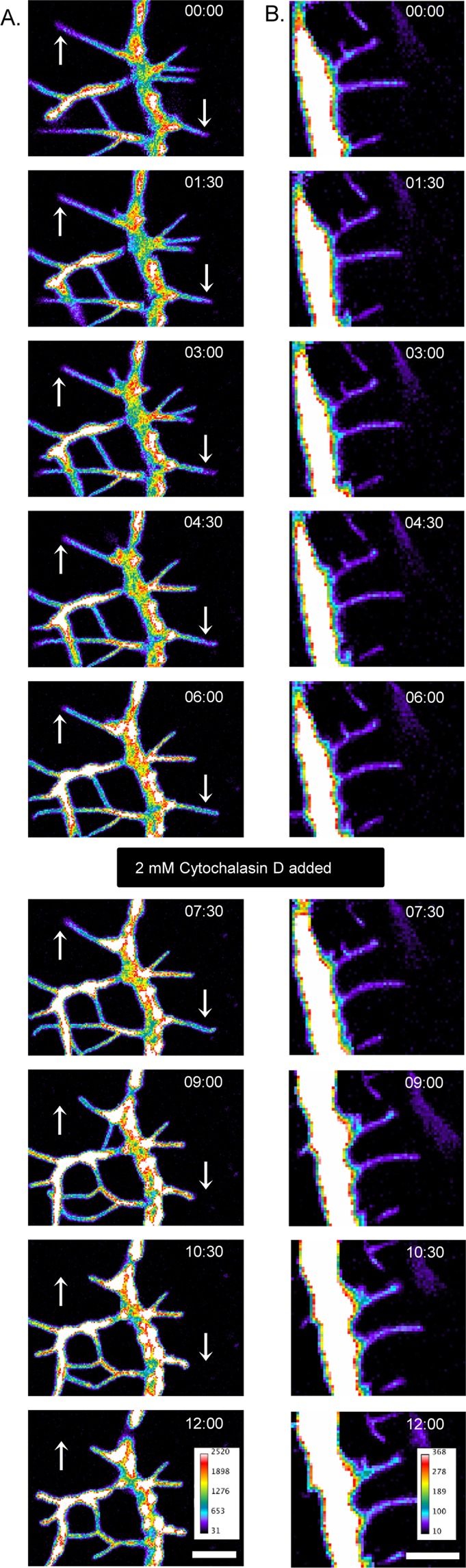
Live cell filopodial dynamics. (**A**) Selected frames of confocal time-lapse of a neurite of N1E-115 cell co-expressing mRFP-IRSp53 (not shown) and GFP-actin (intensity heat map look up table (LUT)). The arrows indicate the dynamic filopodia initially extends before retracting upon addition of Cytochalasin D (at 7 min). Images were recorded every 30 s for a total of 12 min. Scale bar = 5 µm. (**B**) Selected frames of a widefield fluorescence time-lapse showing N1E-115 cell co-expressing mRFP-I-BAR domain of IRSp53 (not shown) and GFP-actin (intensity heatmap LUT). Images were recorded every 30 s for a total of 12 min. The filopodia life time observed (n = 10) was >7 min and was unaffected by the addition Cytochalasin D (at 7 min). Scale bar = 5 µm.

In an attempt to understand the biological role of F-actin in filopodia/protrusive structures generated by IRSp53 and the I-BAR domain, we performed actin de-polymerization experiments by adding 2 mM Cytochalasin D on live cells imaged by time-lapse microscopy. The addition of Cytochalasin D to IRSp53 expressing cells eliminated dynamic filopodia and led to their shortening (Fig. 5A, Cytochalasin D added at 7 minutes, Supplementary Movie S5). By contrast the protrusions in the I-BAR domain of IRSp53 protein expressing cells were not affected by the addition of Cytochalasin D (Fig. 5B and Supplementary Movie S6) showing that actin is essential for IRSp53 filopodial dynamics and their maintenance but not required for the protrusions in the I-BAR expressing cells.

### IRSp53 and its –SH3 domain interactor proteins (Mena, Eps8 and Dynamin) localisation within filopodia

**Figure 6:**
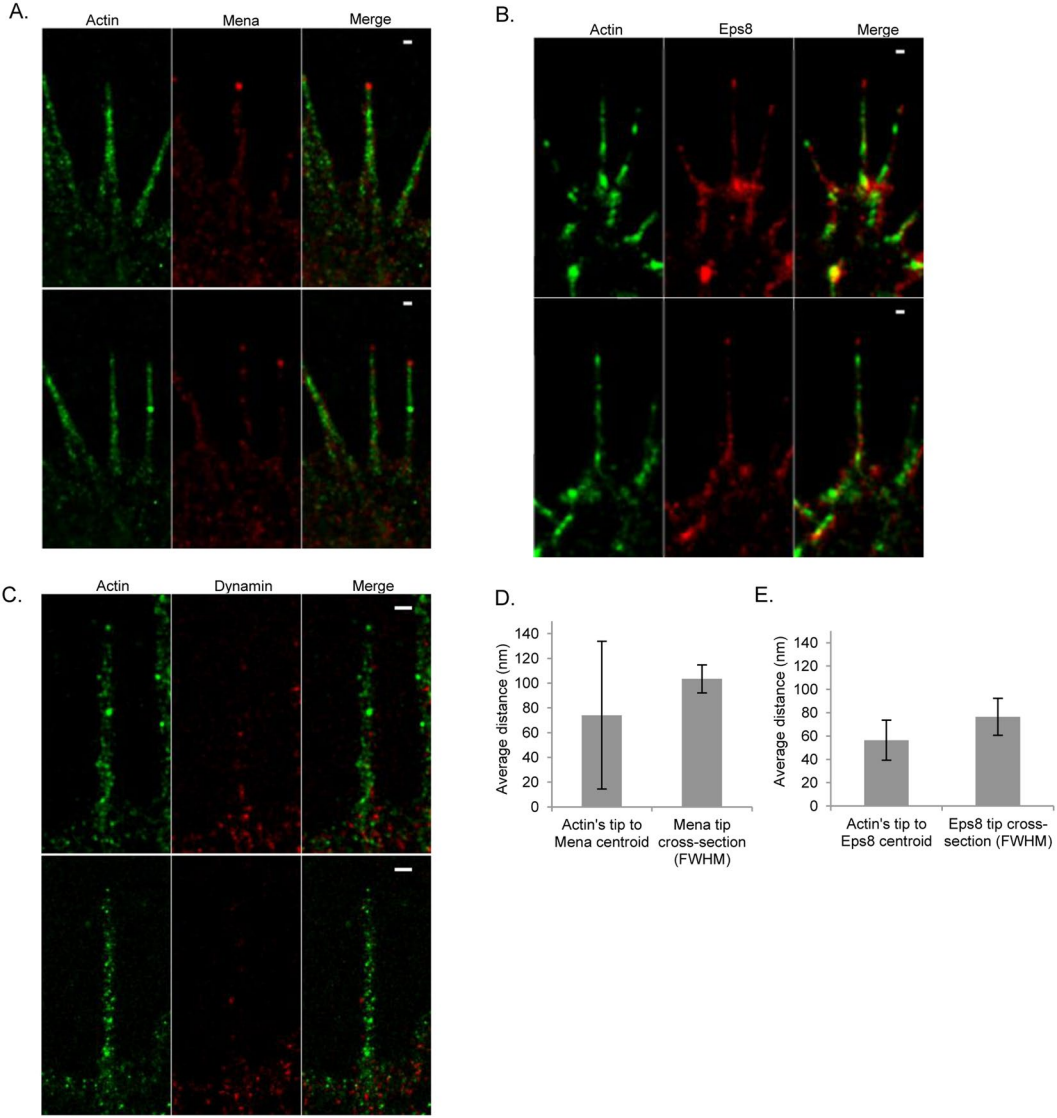
Representative dual colour dSTORM images of filopodia labelled for Mena, Eps8, Dynamin (IRSp53 -SH3 interactors) and actin in N1E-115 cells. (**A**) Filopodia co-expressing GFP-Mena and HA-IRSp53 (unlabelled). Cells are labelled for Mena and endogenous actin using anti-GFP Alexa 647 conjugate and Alexa 568 Phalloidin. (**B**) Filopodia co-expressing GFP-Eps8 and HA-IRSp53 (unlabelled). Cells are labelled for Eps8 and endogenous actin using anti-GFP Alexa 647 conjugate and Alexa 568 Phalloidin. (**C**) Filopodia co-expressing GFP-Dynamin and HA-IRSp53 (unlabelled). Cells are labelled for Dynamin and endogenous actin using anti-GFP Alexa 647 conjugate and Alexa 568 Phalloidin. Representative images of two filopodia images (top and bottom panel) are shown for each protein. Scale bar = 200 nm throughout. (**D**) Distance between Mena centroid to actin tip and FWHM of Mena (n = 12). Measurements were carried out as described in methods and depicted in Supplementary Fig. S6D. (**E**) Distance between Eps8 centroid to actin tip and FWHM of Eps8 (n = 7).

Our previous work has shown that IRSp53’s -SH3 domain interactor proteins (Mena, Eps8 and Dynamin) have time dependent roles and filopodial localisations over the lifetime of filopodia^19^. Briefly, Dynamin during the initiation phase of filopodia formation, Mena during elongation and Eps8 during termination. In order to understand these events further, we generated dual colour 3D-SIM images for filopodia expressing GFP fusions of either Mena, Eps8 and Dynamin along with HA-IRSp53 (not labelled). Endogenous actin was labelled with Alexa 568 Phalloidin (Supplementary Fig. S6A–C). The data shows that Dynamin is localised at the base of filopodia (somewhat diffusely), while Mena and Eps8 at the tips in discrete foci. With the aim of measuring quantitatively the spatial relationships between F-actin, Mena, Eps8, and Dynamin, we performed dual colour dSTORM experiments for similar samples labelled using anti-GFP Alexa 647 conjugate (Fig. 6A–C). The dSTORM of Mena, Eps8 and Dynamin qualitatively supports the 3D-SIM data and earlier findings. Dynamin is predominantly localised to base of filopodia, Mena almost exclusively to the tip, and Eps8 to the tip and shaft (Table 3). Next, we tried to characterise the tip complexes. We measured the distance between Mena, Eps8 and F-actin at the filopodial tips (dSTORM data, refer to depiction of measurement in Supplementary Fig. 6D). The average distances observed were 74.1 ± 59.8 (Mena to actin) and 56.4 ± 17.2 nm (Eps8 to actin) (Fig. 6D,E). We also measured the FWHM of the proteins distribution, as an indicator of the filopodial tip complex size (Fig. 6D,E; Mena ~100 nm, Eps8 ~80 nm).

**Table 3:** IRSp53 and its –SH3 domain interactor proteins (Mena, Eps8 and Dynamin) localisation within filopodia.

Protein Localisation	IRSp53	Mena	Eps8	Dynamin
Tip	+	+++	++	−
Shaft	−	+	+	−
Base	+	−	−	+++
Membrane	++	−	−	−

HA-IRSp53 was coexpressed with either GFP fusions of Mena, Eps8 or Dynamin in duplicate experiments on N1E-115 cells and labelled for respective proteins using anti-GFP Alexa 647 conjugate in one set of experiment. In the other set HA-IRSp53 was labelled with anti-HA-Alexa647. The dSTORM images were obtained as described in experimental methods. The observed protein localisation within filopodia was compared visually and presented. Representing (−) no localisation, (+) being lowest and (+++) highest localisation (n = 5 per protein).

### Membrane association studies using FRAP

**Figure 7:**
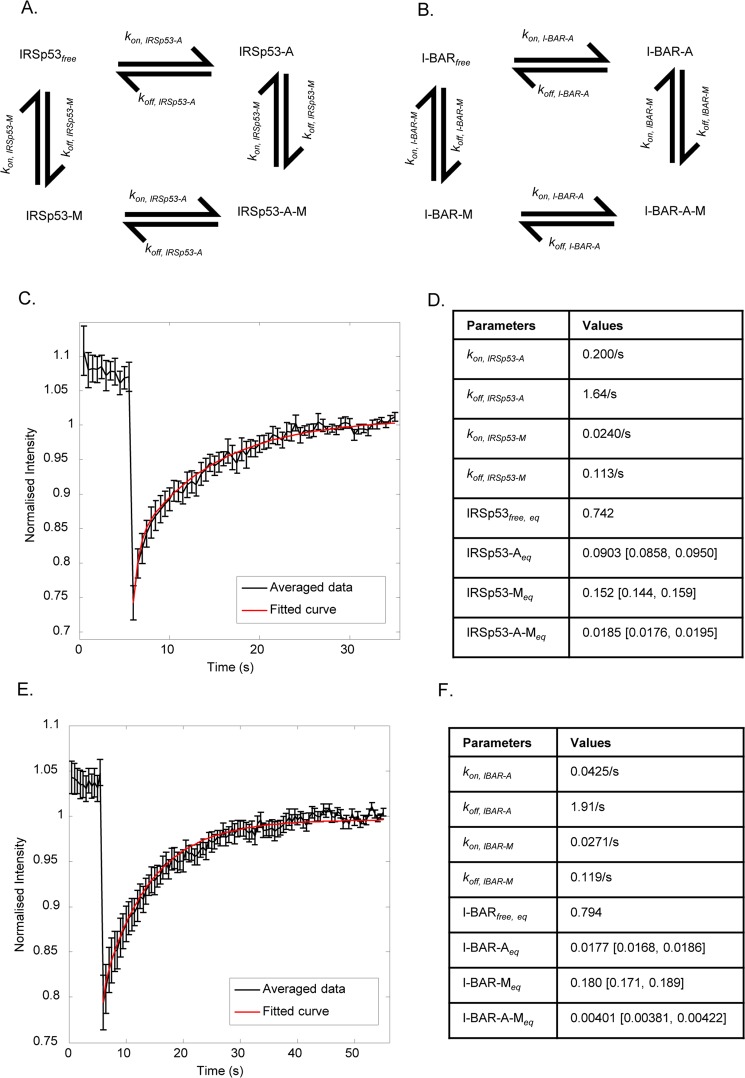
FRAP data describing 4-pool fitting model. (**A**) Schematic of transitions for IRSp53. (**B**) Schematic of transitions for I-BAR mutant. (**C**) The FRAP recovery curve was calculated for each IRSp53 sample and then averaged across (n = 11) samples to produce the black curve. Error bars denote standard error. The red curve shows the fitted curve using the maximum likelihood estimates in (**D**) using the 4-pool model. (**D**) Maximum likelihood estimates of the parameters that best fit the averaged FRAP recovery curve in (**C**). Quantities listed in square brackets denote the minimum and maximum values obtained upon a 5% model parameter perturbation. (**E**) The FRAP recovery curve was calculated for each mutant I-BAR sample and then averaged across (n = 11) samples to produce the black curve. Error bars denote standard error. The red curve shows the fitted curve using maximum likelihood estimates in (**F**) using the 4-pool model. (**F**) Maximum likelihood estimates of the parameters that best fit the averaged FRAP recovery curve in (**E**). Quantities listed in square brackets denote the minimum and maximum values obtained upon a 5% model parameter perturbation.

The protein distribution and kinetic difference between IRSp53 and I-BAR filopodia/protrusions suggests that oligomer stability may play a role.To test this possibility we carried out FRAP^24^ experiments. We assume that the IRSp53 protein can associate with both the plasma membrane and F-actin in the filopodia via F-actin modulators such as Dynamin, Mena, and Eps8, and also exist free. Here, rather than model all these interactions in detail, we instead focus on the relative fractions of the IRSp53 protein that associates with either the plasma membrane or F-actin modulators or both (4-pool model). We use a similar model to analyse I-BAR domain interactions. By matching the recovery curve to a mathematical model where various populations of IRSp53 can transition, we can estimate the association fractions. We assume that there are 4 populations of IRSp53 (i) IRSp53_*free*_: free, i.e., cytosolic; (ii) IRSp53-M: associated with the plasma membrane; (iii) IRSp53-A: associated with the actin bundle; and (iv) IRSp53-A-M: simultaneously associated with both the plasma membrane and the F-actin. In Fig. 7A,B, we show the schematic transitions of proteins IRSp53 and I-BAR domain. In Fig. 7C we show the fitted FRAP recovery curve of IRSp53. By performing maximum likelihood estimation with uniform prior distribution, we were able to estimate the parameters that best reproduces the FRAP recovery curve (n = 11, for both IRSp53 and I-BAR domain). Based on the maximum likelihood estimates, we were able to find the various association of fractions: 15.2% of the IRSp53 is associated with the plasma membrane, 9.03% with the F-actin, 1.85% with both simultaneously, with the remaining being free. These numbers are summarised in Fig. 7D.

Similarly, we conducted FRAP experiments on the fluorescently-tagged I-BAR domain and match the recovery curve to a similar 4-pool model. Based on the maximum likelihood estimates, we were able to find the various association of fractions: 18.0% of the I-BAR domain is associated with the plasma membrane, 1.77% with the F-actin, 0.401% with both simultaneously, with the remaining being free (Fig. 7E). These numbers are summarized in Fig. 7F. Thus, from the FRAP experiments, we observe that about 15% of the IRSp53 proteins are associated with plasma membrane whereas this fraction increases to about 18% for the I-BAR.

To ensure that the fractions obtained from the maximum likelihood estimates do not exhibit huge changes upon parameter deviations, we conduct sensitivity analysis of the model parameters on the protein fractions. The parameters are varied by ±5% and the maximum and minimum fractions are reported in square brackets in Fig. 7D,F. We can observe that the fractions calculated are stable do not deviate too much from the maximum likelihood estimation upon parameter perturbation.

We employed a 4-pool model for the FRAP analysis because a simpler 2-pool model consisting of (i) free protein and (ii) protein bound to membrane cannot reproduce the FRAP recovery curves resulting in a poor fit. The poor fit for IRSp53 using the 2-pool model is shown in Supplementary Fig. S7A,B. Visually, we can observe that the FRAP recovery curve for the 2-pool model is under-fitting at the initial stages after photobleaching from 6 to 16 seconds. The lower R^2^ value for the 2-pool model of 0.855 compared to 0.986 for the 4-pool model (Supplementary Fig.S7C,D) corroborates the under fitting observed. This is confirmed using F-test in which the 4-pool model gives a significantly better fit to the data than the 2-pool model with p < 0.001. This suggests that the 2-pool model is over-simplified to accurately represent the dynamics of IRSp53. On the other hand, a 3-pool model consisting of (i) free protein, (ii) protein bound to membrane and (iii) protein bound to actin neglects the interaction of the protein with both membrane and actin. As a result, a more complicated 4-pool model was chosen since the 4-pool model fits the FRAP recovery curves reasonably and is also representative of the interactions that IRSp53 and IBAR domain undergo.

## Discussions

Previous studies on filopodia mechanisms and dynamics with the IRSp53 pathway were carried out using either confocal or live cell time-lapse microscopy^18^. The resolution of such techniques is limited to ~240 nm making it difficult to understand the protein localisation and architecture of filopodia as they are on a much finer length scale. However recent developments in optical microscopy, particularly the advent of superresolution microscopy, has made it possible to probe the architecture of filopodia. For the first time, to our knowledge, an attempt is made for the quantitative interpretation of filopodia using superresolution microscopy at resolutions down to ~20 nm. This is a challenging undertaking, particularly when studying endogenous proteins with low levels of expression. Meaningful superresolution microscopy has higher requirements for sample preparation (e.g. fixation and suitable mounting so as to preserve ultrastructural details) and quality (e.g. high labelling density, high quality fluorophores, fine tuning imaging buffers, etc.). All the measurements made in this study are based on the expression fusion proteins of either full length IRSp53 protein or I-BAR domain of IRSp53 and endogenous actin. Our previous study^18^ clearly defined the characteristics of filopodia using the data generated from various cell lines with an aim to differentiate from retraction fibers and other cell leading edge protrusions. Here we compared I-BAR family (IRSp53, IRTKS, MIM and ABBA) proteins filopodial characteristics to make a general statement on filopodia formation and to demonstrate the architecture of filopodia using neuroblastoma cell line as a neuronal cell model^18,19,25^.

**Figure 8:**
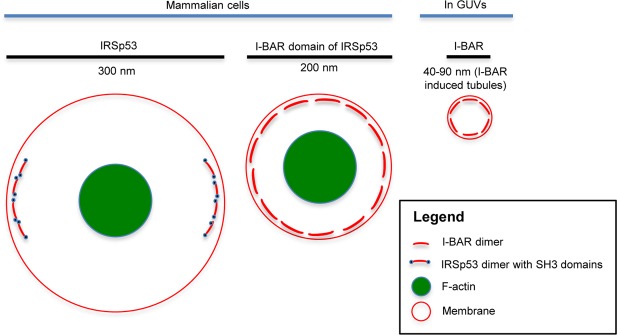
Schematic model of protein localisation and diameter of filopodia/protrusions/tubules generated by IRSp53, I-BAR domain and actin. The model shows the diameter of structures having localisation of IRSp53-actin and I-BAR domain-actin based on the size measured in N1E-115 cell filopodia. The diameter of I-BAR induced tubular size in GUVs is also shown.

The diameter of protrusions generated by I-BAR domain in *in-vitro* experiments using Giant Unilamellar Vesicles (GUV’s) membrane is between 40–90 nm depending on the exact I-BAR domain used^22^. We find the I-BAR protrusions generated in N1E-115 cells are larger with a width of ~200 nm from the FWHM of I-BAR distribution. We assume this arises as a consequence of the presence of F-actin in cells which is absent from the experiments on model GUV membranes. In contrast we find the full-length protein IRSp53 generated filopodia exhibit an even larger width of ~300 nm. To confirm the localisation is not an artifact of HA tagged IRSp53, we verified it by using GFP tagged full length IRSp53 protein. A similar larger width is seen among all the full length I-BAR family proteins, but IRSp53 is distinctly larger. The similar width and distribution was also observed by 3D-SIM. To model this difference we propose a schematic (Fig. 8) of protein distributions. The diameter of F-actin core measured in IRSp53 vs I-BAR protrusions is not dissimilar suggesting it is the membrane associations that leads to these size differences. We observed a 150–200 nm gap between IRSp53 and F-actin from the FWHM measurement, whereas such gap is not present in the I-BAR structures. At the moment we do not have experimental evidence for the reason for the gap between actin and full length IRSp53 protein. We hypothesise that the gap may be resulting from an excluded volume generated by the extended and largely disordered residues of the I-BAR domain. Alternatively there may be a possibility of –SH3 domain interactor protein diffusions within the gap. In any case to confirm either or of both these possibilities requires generation of filopodia with mutant proteins. To this end, we expressed –SH3 mutants of IRSp53 protein in N1E-115 cells. These mutants did not produce any filopodia, which is in agreement with our previous study, showing IRSp53 protein mutants of the –SH3 or CRIB are unable to generate filopodia^18^. Further study will be necessary to understand the origin of the gap and its significance in filopodial biology.

Most interestingly, we observed ‘*spatial segregation*’ of IRSp53 around the lateral edge of filopodial membranes, specifically at the two lateral sides of the filopodia but not on the dorsal and ventral sides, to use anatomical terminology. However the I-BAR domain did not exhibit this spatial segregation and was found throughout the membrane of filopodia. The membrane localisation of I-BAR domain was further verified with plasma membrane targeted fluorescent proteins and plasma membrane specific dyes. This observation is supported by the FRAP data which suggests that a greater proportion of I-BAR domain is associated with membranes in contrast to full length IRSp53. Yang *et al*.^26^ have presented similar data using platinum replica EM; they proposed that I-BAR domain of IRSp53 protein within filopodia are making sub-membranous coat which may support actin free protrusions. Furthermore, Sarrikangas *et al*.^22^ have shown that I-BAR domains binds to the inner leaflet of membrane tubules using model membranes.

The spatial segregation of IRSp53 around filopodial membranes suggests a much more dynamic membrane deformation^27^ induced filopodial structure because larger oligomers are prevented from forming. In contrast, the I-BAR distribution being uniform on membrane is suggestive of an oligomeric structure^28^ with greater membrane deformation activity. Another important observation is that IRSp53 generates diverse membrane morphologies in contrast I-BAR which generates more uniform protrusions. This may again reflect the dominance of oligomers in the latter situation. Similar evidence has emerged from the GUV membrane model studies^22^ suggesting that the I-BAR domain upon binding with membrane making PI(4,5)P_2_ forms lipid clusters. Whereas in the case of full length IRSp53 its spatial segregation to the lateral edges could be explained by the possibility of only binding to membrane with specific curvature^13^. This in turn can be supported by the evidence from studies explaining the IRSp53-Cdc42 binding induced conformational change in full length IRSp53^29,30^ leading to the formation of Cdc42-IRSp53-VASP complex^31^ making it more stable pathway for filopodia extension.

The live cell actin de-polymerization data demonstrates that F-actin is required for the dynamic extension and retraction of filopodia generated by full length IRSp53 but it does not add any significance to the I-BAR generated static protrusions as the filopodial lifetime remained unchanged before and after addition of Cytohalsin D. This data suggests that actin is necessary for the dynamic activity of filopodia in the case of full length IRSp53 and it may be necessary to maintain long term stability in the case of I-BAR protrusions as shown in the Latrunculin B study^26^. In addition, the present actin dependent study is able to differentiate the dynamic filopodia and non-dynamic protrusions in a time dependent way on the same cell. The FRAP data further supports the F-actin association with IRSp53 as seen, the higher percentage of IRSp53 association with actin in contrast to I-BAR domain. The presence of lower percentage of I-BAR domain complex with actin may be due to the existence of actin free transient state in I-BAR induced protrusions due to the generation of sub-membranous coat^26^, whereas the higher percentage of actin-IRSp53 complex may also explain the presence of actin in the filopodial tips^32,33^. The 3D-SIM and dSTORM data of Mena, Eps8 and Dynamin confirms and extend previous observations of tip complexes^16,19^. IRSp53 thus represents a link between membrane deformation and F-actin dynamics through these proteins. Further work will be required to understand what controls the recruitment of these -SH3 domain interactors during the lifetime of filopodia.

In conclusion, by using superresolution localisation microscopy we have shown distinct localisations of IRSp53 and its I-BAR domain proteins within filopodia. The I-BAR domain generates non-dynamic protrusions, its localisation is uniform, and we reason that this is because of the formation of stable I-BAR domain oligomers. In contrast, the localisation of IRSp53 is spatially segregated along the lateral edges of the filopodial membrane, and non-uniform, stable oligomers are unlikely to form making the protrusions dynamic. Taken together, the data suggest that the low stability of IRSp53 association with membranes, along with its ability to deform membranes, and recruit actin modulators, plays a role in the molecular mechanism of filopodia formation.

## Methods

### Plasmids, antibodies and reagents

mRFP-IRSp53, GFP-actin, HA-IRSp53, GFP-I-BAR (domain of IRSp53) and GFP-actin plasmids were made as described previously^18^. pEGFP-IRSp53 was a gift from Prof. Akiko Yamagishi, National Cardiovascular Center Research Institute, Japan. mRFP-I-BAR was made by replacing GFP with mRFP in pXJ40-GFP vector. GFP-Mena was a gift from Prof. Klemens Rottner (Helmholtz Center for Infection Research, Germany). eGFP-Eps8 was a kind gift from Prof. Giorgio Scita (FRIC Institute of molecular Oncology foundation-European Institute of Oncology, Italy). pGFP-N1-human Dyn1 was a gift from Prof. Pietro De Camilli (Yale University). IRTKS, MIM and ABBA in pGFP-N1 vector were kind gift from Prof. Pekka Lappalainen, Institute of Biotechnology, Finland. Human Pinkbar (GenBank BC015619) template was purchased from PlasmID repository at Harvard Medical School and sub-cloned into pXJ40-EGFP vector. I-BAR-GFP of MIM, ABBA, IRTKS and Pinkbar were generated by making fusions of respective I-BAR domains in pXJ40-GFP vector. pIRESpuro3-mCherry–ABP 140p was a gift from Prof. Philippe Chavrier (CNRS, France). Plasma membrane targeted YFP (PMT-YFP) was generated as reported previously^34^.

### Materials

Anti-HA-tag-Alexa fluor 647 conjugate was purchased from Medical and Biological laboratories company, Japan. Anti-chicken Alexa 488 secondary (Goat), anti-rabbit Alexa 568 (Goat) secondary, Polyclonal anti-GFP Alexa fluor 647 conjugate, Alexa fluor 568 and 647 Phalloidins, Wheat germ agglutinin (WGA) Alexa fluor 594 plasma membrane stain, CellMask (CM) Orange plasma membrane stain, cell culture medium and reagents were purchased from Thermo Fisher Scientific, Singapore. Polyclonal anti-RFP primary (rabbit) antibody was obtained from Rockland Immunochemicals. Polyclonal anti-GFP (chicken) antibody was purchased from Cell Signaling Technology. Cytochalasin D, Laminin and Mercaptoethylamine (MEA) were obtained from Sigma-Aldrich, Singapore. Coverslips with embedded gold fiducials were purchased from Hestzig LLC, USA. VECTASHIELD (H-1000 without DAPI) mounting medium was obtained from Vector Laboratories. Lab-Tek dish, Lipofectamine 2000 and TetraSpeck beads were purchased from Thermo Fisher Scientific, Singapore. MatTek dish was obtained from Research Support Center, A*STAR, Singapore. Jetprime (Polyplus-transfection) reagent was purchased from Axil Scientific, Singapore.

### Cell culture

Mouse neuroblastoma N1E-115 and Hela cells were grown in high glucose DMEM medium having 10% serum 1% penicillin/streptomycin in a humidified 37 °C incubator with 5% CO_2._ The N1E-115 cells were split into pre-cleaned, Laminin coated glass bottom 35 mm MatTek dish (for live cell imaging)/Laminin coated 8 well Lab-Tek II dish (for single colour dSTORM)/embedded coverslips with gold fiducials (for dual colour dSTORM) and allowed to grow for overnight in 37 °C with 5% CO_2_ incubator. The following day cells were transiently transfected with respective cDNA in serum starving condition^18^ using Jetprime (Polyplus-transfection) and incubated in 37 °C with 5% CO_2_ incubator for another 4 h in serum free medium. The medium was then replaced with 5% serum containing medium and incubated further for overnight in 37 °C with 5% CO_2_ incubator.

### Scoring for filopodia and protrusions

Filopodia characterisation was performed on N1E-115 cells co-expressing respective I-BAR fusion proteins along with ABP- mCherry (actin binding protein). Live cell imaging was carried out on Olympus IX83 wide field fluorescence microscope fitted with incubator (37 °C and 5% CO_2_). The system was equipped with a light source of mercury burner and Coolsnap HQ2 CCD camera (Photometrics). The images were acquired at 10 s intervals using Metamorph software (Molecular Devices) for both fluorescence and DIC mode. Filopodia/protrusion dynamic activities were scored as described previously^18^. Briefly, filopodia lifetime was defined as the difference in time measured between appearance and disappearance of filopodia. The length was measured from the base to tip of filopodia. The experiment was repeated three times and representative data from one experiment is given.

### Sample preparation for plasma membrane specific dye label and imaging

N1E-115 cells were grown on Laminin coated 22 × 22 mm coverslips and transfected with either PMT-YFP or GFP-I-BAR. On the following day cells were rinsed with PBS and incubated with phenol red free DMEM medium containing 0.5x CellMask orange 555 for 8 min. Cells were further washed and fixed in 4% para-formaldehyde and mounted using VECTASHIELD (H-1000 without DAPI) on glass slides followed by three washing. For WGA-594 plasma membrane specific dye labelling, the transfected cells were fixed and treated with WGA-594 label (1:200) for 10 min in PBS and mounted followed by three washing in PBS. Image acquisition was carried out on Olympus FV1000 microscope and processed the data using ImageJ software for intensity overlapping measurement. The extracted intensity data was normalized and plotted in Excel.

### Actin dependency assay

N1E-115 cells were co-transfected with either mRFP-IRSp53 or mRFP-I-BAR and GFP-actin. The following day cells were subjected to time lapse imaging on Olympus FV1000 confocal / IX83 wide field fluorescence microscope fitted with incubator (37 °C and 5% CO_2_). Images were acquired using EMCCD camera and MetaMorph software. Cytochalasin D (2 mM) was added 7 min after initiating the imaging and followed for another 10 min. Image processing was done using ImageJ software. The experiment was repeated three times and a representative image is given.

### Sample preparation for single colour dSTORM imaging

Transiently transfected N1E-115 cells with either of –HA tag or GFP fused plasmids were washed and fixed using 4% para-formaldehyde. Cells were permeabilised using 0.5% Triton x-100 in PBS for 10 min and blocked using 5% BSA for 30 min followed by a PBS rinse. Cells were labelled for 60 min either with anti-HA Alexa 647 or anti-GFP Alexa 647 conjugate (1:100) in PBS buffer for respective fusion proteins. Endogenous actin was labelled using Alexa 647 labeled Phalloidin (1:100) in place of antibody for 60 min. Cells were thoroughly washed further in PBS containing 0.1% Tween 20. Single colour dSTORM imaging was performed in freshly prepared 100 mM MEA pH 8.4^35^.

### Sample preparation for dual colour dSTORM imaging

Dual colour imaging sample was prepared using Laminin coated gold fiducials embedded coverslips for drift correction. Transiently transfected N1E-115 cells were labelled with respective Alexa 647 (1:100) antibody conjugate and Alexa 568 labelled Phalloidin (1:200) for endogenous actin. Dual colour dSTORM imaging was performed using 100 mM MEA at pH.8.0.

### Sample preparation for two colour 3D SIM imaging

N1E-115 cells were grown on Laminin coated 18 × 18 mm coverslip (superresolution microscopy grade #1.5 H) and transfected with HA-IRSp53. Cells were labelled with anti-HA Alexa 647 and Alexa 568 Phalloidin (endogenous actin). In the case of GFP fusions of Mena, Eps8 and Dynamin, transfected cells were used without any further staining. Endogenous actin was labelled with Alexa 568 Phalloidin. Cells were further washed and mounted using VECTASHIELD (H-1000 without DAPI). Excess mounting medium was carefully removed and sealed using nail polish.

### Sample preparation for thee colour 3D SIM imaging

N1E-115 cells grown on Laminin coated 22 × 22 mm (#1.5 H) coverslips were cotransfected with PMT-YFP and mRFP-I-BAR/IRSp53. Following day cells were washed and fixed with 4% para-formaldehyde and quenched further with 200 mM NH_4_Cl. Cells were further washed for three times and permeabilised using 0.5% Triton x-100 in PBS for 10 min. Cells were blocked using 5% BSA for 30 min followed by a PBS rinse. Cells were stained with primary antibodies of anti-GFP (1:100) for PMT-YFP and anti-RFP (1:100) for I-BAR/IRSp53 for overnight at 4 °C. The primary antibody stained cells were further washed three times and labelled with respective secondary antibodies of Alexa 488 ((1:200; for PMT-YFP) and Alexa 568 (1:200; for I-BAR/IRSp53) for 60 min. Endogenous actin was labelled with Alexa 647 Phalloidin (1:100) along with secondary antibodies. The cells were further washed for three times in PBS (containing 0.1% Tween 20) and mounted on clean cover slides using VECTASHIELD (H-1000 without DAPI).

### dSTORM microscope setup

The single colour dSTORM imaging was done using in-house built system (Supplementary  Fig. S1). It is a free space coupled TIRF (Total Internal Reflection Fluorescent) configuration microscope built on Olympus IX 71 inverted fluorescence microscope. The inverted microscope included bright field optics to find the cell of interest and a 1.6 x intermediate magnification. A high numerical aperture Olympus TIRF objective oil immersion lens, 100x/1.49 was used for single molecule imaging. The system included Olympus IX2-NPS nosepiece stage that significantly reduces lateral and axial drifts. The illumination side of the system included Coherent 405 nm (100 mW) and TOPTICA 640 nm (150 mW) lasers for activation and imaging. Lasers were merged using the appropriate dichroic mirrors and passed through the Acousto-optic tunable filter (AOTFnC-VIS, AA Opto Electronic) for wavelength selection and power adjustment. The AOTF was controlled by laser control software developed by QuickPALM^36^ developers. A 100 mm focusing lens and 400 mm collimating lens (Thorlabs) was used to expand the beam 4 times to give sufficient power to send most of the molecules to the OFF state. A mirror placed in between these two lenses was conjugated to the back focal plane of the objective lens to provide TIRF illumination. A 200 mm focusing lens was then used to focus the laser on to the back focal plane of the objective lens. For detection a filter wheel (Sutter Instruments) consisting of Semrock 736/128 nm BrightLine emission filter was used to detect Alexa 647 fluorescence. The Evolve liquid cooled 512 × 512 EMCCD camera (Photometrics) was used for efficient detection of the single fluorescent molecules.

dSTORM image data acquisition was performed using Metamorph acquisition software v7.8. The exposure time was set to 15 ms at an EM gain of 200. The 640 nm laser at low power in combination with the Semrock quad band filter cube (Di01-R405/488/561/640) was used to scan the sample in TIRF mode to identify cells that were labeled with the Alexa 647 Phalloidin. Only the centre quadrant of the EMCCD chip was used for imaging (256 × 256) which offered a high frame rate of 60 frames/s. The 640 nm laser power was further increased using the laser control software^36^ until it was sufficient to send most of the fluorescent molecules to the dark state. In time-lapse image acquisition mode a total of 10,000–15,000 frames were recorded. The sample was initially only illuminated with 640 nm laser, however after a few thousand frames when the number of blinks reduces a short pulse of 405 nm at very low power (µW) was used to bring more molecules back to the bright state. Single colour dSTORM image reconstruction was performed using *rapidSTORM*^37^(http://www.superresolution.de) software, an open source tool for processing single molecule localisation data using 2D Gaussian fitting. The full width at half maximum of point-spread function was set to 320 nm. This was measured by using 100 nm TetraSpeck beads at 640 nm excitation. The computation for single molecule fitting and reconstruction was performed using the software.

### SIM image data acquisition

SIM imaging was carried out on a DeltaVision OMX v4 Blaze microscope (GE Healthcare) equipped with 488 nm 568 nm and 647 nm lasers and a BGR-FR filter drawer (emission wavelengths 528/48 for Alexa 488, 609/37 for Alexa 568 and 683/40 for Alexa 647), a Plan Apochromat 100x/1.4 PSF oil immersion objective lens (Olympus) using an oil of 1.514 refractive index, and individual Evolve liquid-cooled EM-CCD cameras (Photometrics). A total of 15 images were acquired per section per channel (comprising 3 rotations and 5 phase shifts of the structured illumination pattern) at a z-spacing of 0.125 μm^38,39^. Structured illumination reconstruction and alignment were carried out using the SoftWoRx (GE Healthcare) program. For processing three colour 3D SIM cross-sectional view image processing Bitplane Imaris software (Oxford Instruments Company) was used.

### Two colour dSTORM imaging

DeltaVision OMX v4 Blaze microscope equipped with optical components described earlier and Olympus Plan Apochromat 100X/1.49 TIRF oil immersion objective lens along with galvo-driven ring TIRF module for sequential two-channel TIRF imaging with different critical angles was used. Cells labelled with Phalloidin 568 and Alexa 647 were excited with appropriate lasers. The 405 nm laser at very low power (µW) was used to bring more molecules back to the bright state. Beam concentrator was used in this mode to generate higher laser power for single molecule blinking. Images were captured with a liquid-cooled Photometrics Evolve EM-CCD camera for each channel. Exposure time 20 ms, laser power and camera EM gain were all adjusted to achieve maximum photo blinking of the respective fluorescent dyes. A total of 10000 to 20000 frames were recorded.

The superresolution image was generated from raw OMX “.dv” data using ThunderSTORM^40^ ImageJ plugin. A custom ImageJ macro that uses ThunderSTORM plugins was also written to batch process the large amount of raw OMX “.dv” data (photoelectrons per A/D set to 0.067). The output images from batch processing were evaluated based on quality of localisation and fiducial markers. Those data sets that passed the qualitative visual inspection check was then re-processed using ThunderSTORM with the actual photoelectrons per A/D value. This filter helped to keep localisation uncertainty to less than 40 nm and apply drift correction to generate Gaussian normalized superresolution images. Lastly, ImageJ plugin bUnwarpJ^41^ was used to correct for the chromatic alignment between channels using fiducial markers as reference.

### FWHM measurement

For analysis of superresolution images, a custom written macro in FIJI (ImageJ)^42^ was used (Supplementary Fig. S2D). For each filopodia/protrusion, we defined a region of interest (ROI) which is a line from the base to the tip of the filopodia. Based on this ROI, the macro will generate 11 evenly spaced perpendicular sampling lines from base to tip of filopodia. The relative position of the sampling lines is 0 at base and 1 at the tip. Those sampling lines were then added to ROI manager by macro and used to extract intensity values from images either for single Gaussian fitting to calculate the FWHM or double Gaussian fitting for peak to peak distance + half left FWHM + half right FWHM. In the case of actin images, the extracted intensities were used to calculate FWHM by single Gaussian fitting. For HA-IRSp53 images, the extracted intensities were used for double Gaussian fitting to calculate peak to peak distance + half left FWHM + half right FWHM and for the I-BAR, those sampling lines were used for manual measurement of the width. The distances between tip of filopodia to Eps8 and tip of filopodia to Mena were also measured manually (Supplementary Fig. S6D).

### FRAP setup

FRAP experiment was performed on a motorized Nikon Ti inverted microscope equipped with the Perfect Focus System (Nikon, Japan) using a Plan Apo 60x/1.4 oil immersion objective lens, 491 nm laser (iLas2 FRAP-3D; Roper Scientific, France), CSU-22 spinning disk confocal scanning head (Yokogawa Electric Corp., Japan), and an Evolve EM-CCD camera (Photometrics, USA). Transiently transfected (using Lipofectamine 2000) Hela cells expressing GFP-IRSp53 and GFP-I-BAR (IRSp53 mutant) cultures were prepared in 35 mm glass bottom Willco dishes(WillCo Wells, The Netherlands) and maintained at 37 °C in 5% CO_2_ humidified environment using an on-stage incubator (Chamlide, Live Cell Instruments, S. Korea). Time-lapse image acquisitions were controlled using MetaMorph software (150 ms exposure, EM-gain 300) and FRAP setting was controlled through the iLas2 control window. The bleaching was carried out on filopodia with a rectangular region using 100% laser power and imaged with 15% laser power. Five pre-bleach images at 0.5 s interval was recorded before FRAP bleaching. The FRAP recovery was recorded for another 90 frames at every 0.5 s.

### FRAP Analysis

Individual FRAP recovery samples were first normalized such that their final recovery fraction is 1. These individual recovery curves were then averaged across samples to produce the averaged FRAP recovery curves. We fit the averaged FRAP recovery curve to the 4-pool model. To obtain the parameters for the 4-pool model, we perform the following parameter estimation.

#### Parameter estimation for transitions involving IRSp53

Ordinary differential equations can be written for the transitions listed in IRSp53. These are

$$\begin{array}{rcl}\frac{d{{\rm{IRSp53}}}_{free}}{dt} & = & \begin{array}{c}-k{\ast }_{on,IRSp53 \mbox{-} A}({{\rm{IRSp53}}}_{free})({\rm{A}})+{k}_{off,IRSp53 \mbox{-} A}({\rm{IRSp53}} \mbox{-} {\rm{A}})\\ \,-k{\ast }_{on,IRSp53 \mbox{-} M}({{\rm{IRSp53}}}_{free})(M)+{k}_{off,IRSp53 \mbox{-} M}({\rm{IRSp53}} \mbox{-} {\rm{M}})\end{array}\\ \frac{d{\rm{IRSp53}} \mbox{-} {\rm{A}}}{dt} & = & \begin{array}{c}k{\ast }_{on,IRSp53 \mbox{-} A}({{\rm{IRSp53}}}_{free})({\rm{A}})-{k}_{off,IRSp53 \mbox{-} A}({\rm{IRSp53}} \mbox{-} {\rm{A}})\\ \,-k{\ast }_{on,IRSp53 \mbox{-} M}(\mathrm{IRSp53} \mbox{-} A)(M)+{k}_{off,IRSp53 \mbox{-} M}({\rm{IRSp53}} \mbox{-} {\rm{A}} \mbox{-} {\rm{M}})\end{array}\\ \frac{d{\rm{IRSp53}} \mbox{-} {\rm{M}}}{dt} & = & \begin{array}{c}k{\ast }_{on,IRSp53 \mbox{-} M}({{\rm{IRSp53}}}_{free})(M)-{k}_{off,IRSp53 \mbox{-} M}({\rm{IRSp53}} \mbox{-} {\rm{M}})\\ \,-k{\ast }_{on,IRSp53 \mbox{-} A}(\mathrm{IRSp53} \mbox{-} M)({\rm{A}})+{k}_{off,IRSp53 \mbox{-} A}({\rm{IRSp53}} \mbox{-} {\rm{A}} \mbox{-} {\rm{M}})\end{array}\\ \frac{d{\rm{IRSp53}} \mbox{-} {\rm{A}} \mbox{-} {\rm{M}}}{dt} & = & \begin{array}{c}k{\ast }_{on,IRSp53 \mbox{-} M}(\mathrm{IRSp53} \mbox{-} A)(M)-{k}_{off,IRSp53 \mbox{-} M}({\rm{IRSp53}} \mbox{-} {\rm{A}} \mbox{-} {\rm{M}})\\ \,+k{\ast }_{on,IRSp53 \mbox{-} A}(\mathrm{IRSp53} \mbox{-} M)({\rm{A}})-{k}_{off,IRSp53 \mbox{-} A}({\rm{IRSp53}} \mbox{-} {\rm{A}} \mbox{-} {\rm{M}})\end{array}\end{array}$$

Since bleaching affects only the visible free and complexed molecules, free binding sites on actin and membrane do not change following bleaching. Furthermore, since the bleached area of the filopodia is extremely small compared to the size of the cell, we can assume that IRSp53_free_ instantly equilibrates after the bleach and its concentration remain unchanged throughout the recovery process. Thus we can simplify the above equations using the following relations:

$$\begin{array}{rcl}{k}_{on,IRSp53 \mbox{-} A} & = & k{\ast }_{on,IRSp53 \mbox{-} A}({\rm{A}})\\ {k}_{on,IRSp53 \mbox{-} M} & = & k{\ast }_{on,IRSp53 \mbox{-} M}(M)\\ IRSp{53}_{free,eq} & = & IRSp{53}_{free}\end{array}$$

The original differential equations are thus simplified to

$$\begin{array}{rcl}\frac{d{\rm{IRSp53}} \mbox{-} {\rm{A}}}{dt} & = & \begin{array}{c}{k}_{on,IRSp53 \mbox{-} A}({{\rm{IRSp53}}}_{free,eq})-{k}_{off,IRSp53 \mbox{-} A}({\rm{IRSp53}} \mbox{-} {\rm{A}})\\ \,-{k}_{on,IRSp53 \mbox{-} M}(\mathrm{IRSp53} \mbox{-} A)+{k}_{off,IRSp53 \mbox{-} M}({\rm{IRSp53}} \mbox{-} {\rm{A}} \mbox{-} {\rm{M}})\end{array}\\ \frac{d{\rm{IRSp53}} \mbox{-} {\rm{M}}}{dt} & = & \begin{array}{c}{k}_{on,IRSp53 \mbox{-} M}({{\rm{IRSp53}}}_{free,eq})-{k}_{off,IRSp53 \mbox{-} M}({\rm{IRSp53}} \mbox{-} {\rm{M}})\\ \,-{k}_{on,IRSp53 \mbox{-} A}({\rm{IRSp53}}-{\rm{M}})+{k}_{off,IRSp53 \mbox{-} A}({\rm{IRSp53}} \mbox{-} {\rm{A}} \mbox{-} {\rm{M}})\end{array}\\ \frac{d{\rm{IRSp53}} \mbox{-} {\rm{A}} \mbox{-} {\rm{M}}}{dt} & = & \begin{array}{c}{k}_{on,IRSp53 \mbox{-} M}(\mathrm{IRSp53} \mbox{-} A)-{k}_{off,IRSp53 \mbox{-} M}({\rm{IRSp53}} \mbox{-} {\rm{A}} \mbox{-} {\rm{M}})\\ \,+{k}_{on,IRSp53 \mbox{-} A}(\mathrm{IRSp53} \mbox{-} M)-{k}_{off,IRSp53 \mbox{-} A}({\rm{IRSp53}} \mbox{-} {\rm{A}} \mbox{-} {\rm{M}})\end{array}\end{array}$$

We then perform maximum likelihood estimation. The uniform range of (0, 2) was used for the prior distributions of the parameters *k*_*on*, *IRSp53-A*,_ *k*_*on*, *IRSp53-M*,_ *k*_*off*, *IRSp53-A*_ and *k*_*off*, *IRSp53-M*._

The procedure for the maximum likelihood estimation is as follows:

1. From the uniform prior distribution, randomly select a new set of parameter values for *k*_*on*, *IRSp53-A*,_ *k*_*on*, *IRSp53-M*,_ *k*_*off*, *IRSp53-A*_ and *k*_*off*, *IRSp53-M*_.
2. Using the selected parameter values in step 1, the differential equations for IRSp53-A, IRSp53-M and IRSp53-A-M can be simulated in MATLAB using the function ode45. Initial values for IRSp53-A, IRSp53-M and IRSp53-A-M are set to 0 immediately following bleaching. The FRAP recovery curve can then be obtained by summing up the contributions from IRSp53_*free*_, IRSp53-A, IRSp53-M and IRSp53-A-M over time.
3. Total error between the simulated FRAP recovery curve and the averaged FRAP recovery data is recorded.

Steps 1 to 3 are repeated for 20 million iterations and the set of parameters that gave the lowest error was plotted as the fitted curve.

For I-BAR, we use the same procedure to fit the averaged I-BAR FRAP recovery curve to the 4-pool model.

### Statistical analysis

Three independent experiments were carried out to generate quantitative data and is expressed as mean ± s.d. Microsoft Excel was used to calculate s.d.

## Supplementary information

Supplementary Info

Supplementary movie S1

Supplementary movie S2A

Supplementary movie S2B

Supplementary movie S3

Supplementary movie S4

Supplementary movie S5

Supplementary movie S6

## Data Availability

Data sets are available from authors upon request.
