# Supplementary material for: Superresolution microscopy reveals distinct localisation of full length IRSp53 and its I-BAR domain protein within filopodia
Source: Sci Rep. 2019 Feb 21;9:2524. doi: 10.1038/s41598-019-38851-w (PMC6385187; doi:10.1038/s41598-019-38851-w)
Supplement: Supplementary file 1 — Supplementary Info [file 41598_2019_38851_MOESM1_ESM.pdf]

Superresolution microscopy reveals distinct localisation of full length IRSp53 and its I-BAR domain protein within filopodia

Thankiah Sudhaharan[1,2*], Srivats Hariharan[1,†], John Soon Yew Lim[1,2], Jaron Zhongliang Liu[1,‡], Koon Yen Ling[3], Graham D. Wright[1,2], Keng Hwee Chiam[3], Sohail Ahmed[1,*]

Affiliations
[1]Institute of Medical Biology, A*STAR, Singapore 138684, Singapore
[2]Skin Research Institute of Singapore, A*STAR, Singapore138648, Singapore
[3]Bioinformatics Institute, A*STAR, Singapore 138671, Singapore
[†]Present address: Olympus Singapore Pte Ltd, Singapore 248373, Singapore
[‡]Present address: GE Healthcare, Singapore 099253, Singapore

[*]To whom correspondence and request for materials should be addressed to T.S. (e-mail: sudhaharan.thankiah@sris.a-star.edu.sg) or S.A. (e-mail: hambalt116@gmail.com)

CONTENTS

Supporting information list pdf file

Supplementary Fig. S1.
Supplementary Fig. S2.
Supplementary Fig. S3
Supplementary Fig. S4.
Supplementary Fig. S5.
Supplementary Fig. S6.
Supplementary Fig. S7.
Supplementary movie legends

Supporting information movie with MP4 movie file format

Supplementary movie S1.
Supplementary movie S2 A.
Supplementary movie S2 B.
Supplementary movie S3.
Supplementary movie S4.
Supplementary movie S5.
Supplementary movie S6.

Figure S1

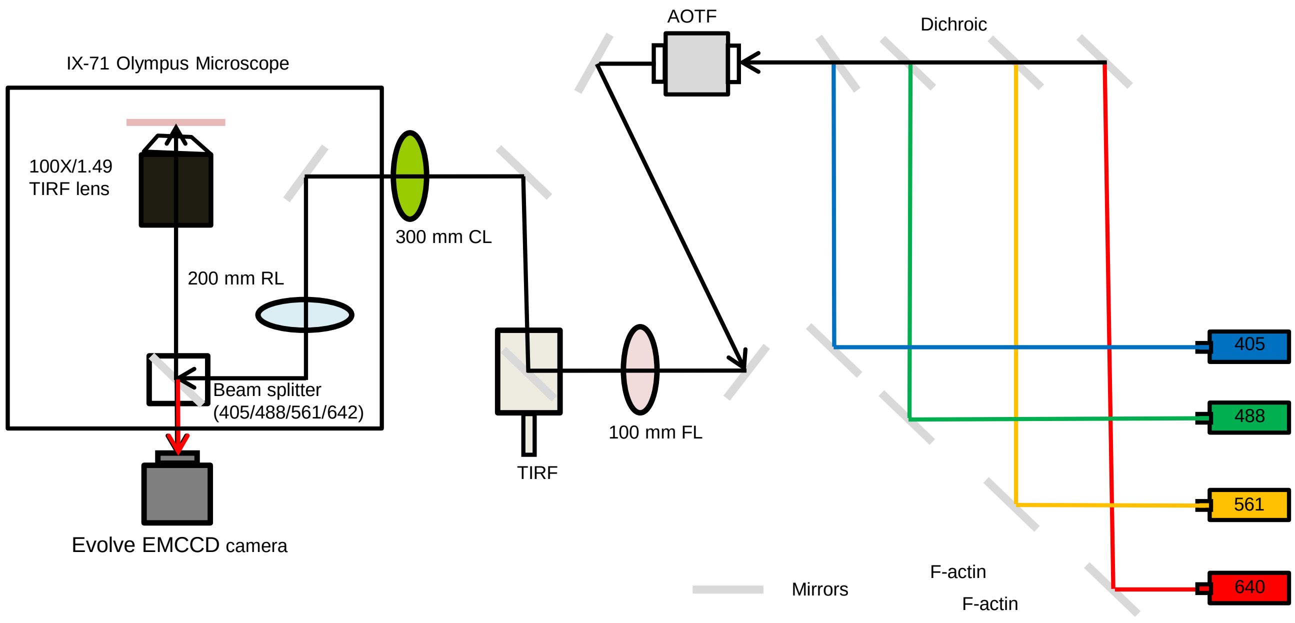

Supplementary Fig. S1.

Schematics of the in-house free space coupled TIRF system used for dSTORM imaging. The TIRF Olympus IX 71 inverted fluorescence microscope system was equipped with four lasers (405,488,561and 640 nm), high numerical aperture Olympus TIRF 100 x/1.49 oil immersion objective and Evolve EMCCD camera with necessary optical components for light path. FL; focusing lens, CL; collimating lens, RL; relay lens, and AOTF; acousto-optic tunable filter.

Figure S2

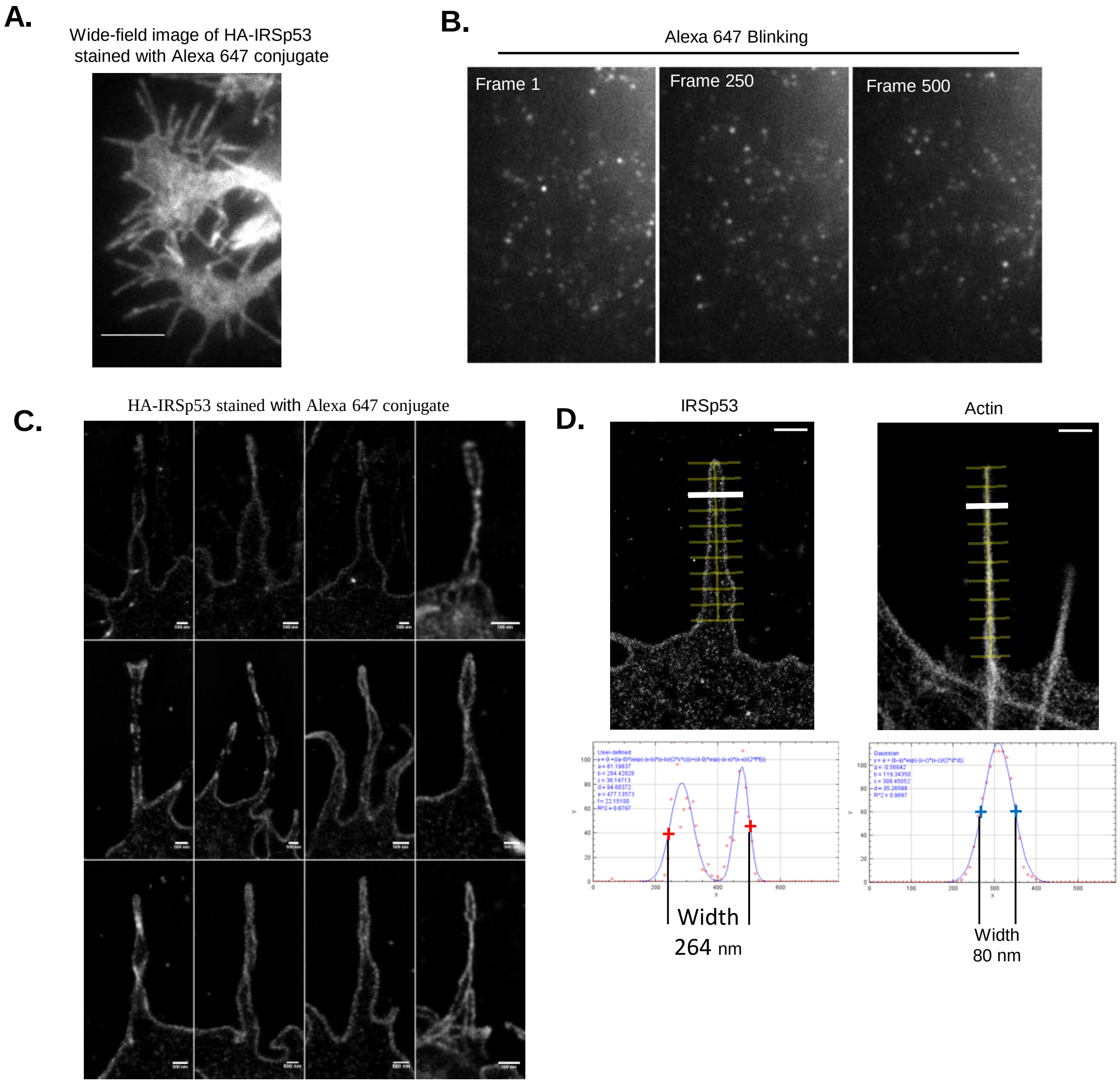

Supplementary Fig. S2.

(A) Shown is representative ROI wide-field image of HA-IRSp53 expressing N1E-115 cell used for dSTORM imaging. (B) The single molecule blinking pattern of anti-HA Alexa 647 conjugate staining HA-IRSp53 of the ROI shown in (A) at different frame points (scale bar = 5 µm). The experimental details are described in methods section. (C) Montage of single colour dSTORM images showing various shapes of HA-IRSp53 expressing filopodia labelled with anti-HA-Alexa 647 conjugate in Hela cells. (D) Methodology for determining FWHM/width measurements used for Fig. 3. A FIJI macro automatically generated 11 lines perpendicular to the filopodia/protrusion and an intensity profile was plotted for each. Single (actin) or double Gaussian (IRSp53) fitting was applied and the FWHM determined. For double Gaussian fitting the width of the filopodia/protrusion was measured as the distance between the two outer FWHM points (i.e. furthest left to furthest right; + to +), whereas for single Gaussian it was simply the FWHM (+ to +). Scale bar = 500 nm throughout.

Figure S3

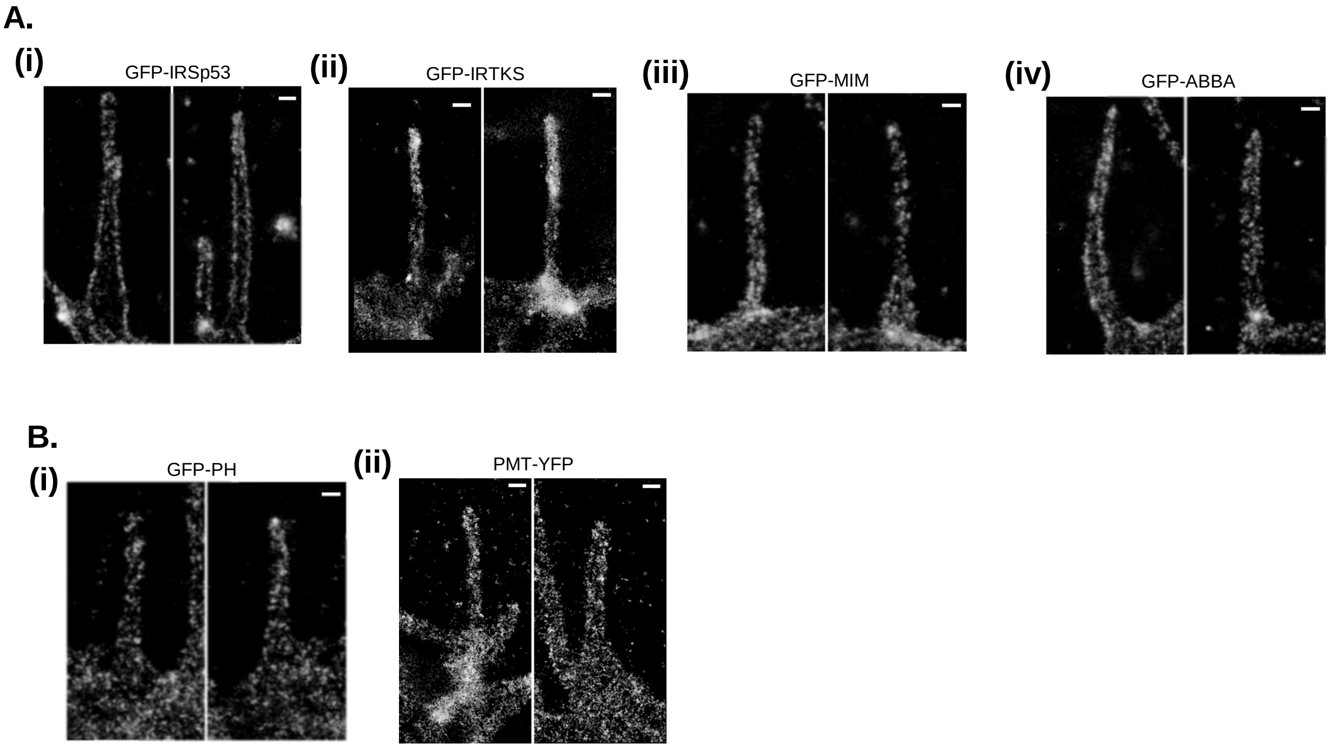

Supplementary Fig. S3.

(A). Representative single colour dSTORM images of filopodia expressing respective full length I-BAR family proteins fused with GFP and stained with anti-GFP Alexa 647 conjugate in N1E-115 cells.

(i).pEGFP-IRSp53 (ii) GFP-IRTKS (iii) GFP-MIM (iv) GFP-ABBA. Two images are shown from each protein, for details of data processing see methods section (scale bar = 200 nm).

(B). dSTORM filopodia control image of cells expressing membrane localisation proteins (targeted controls fused with GFP) stained with anti-GFP Alexa 647 conjugate in N1E-115 cells.

(i).GFP-PH (pleckstrin homology domain of PLC protein) (ii) PMT-YFP (Plasma membrane targeted sequence fused with YFP). Representative two images are shown from each protein for details of data processing see methods section (scale bar = 200 nm).

Figure S4

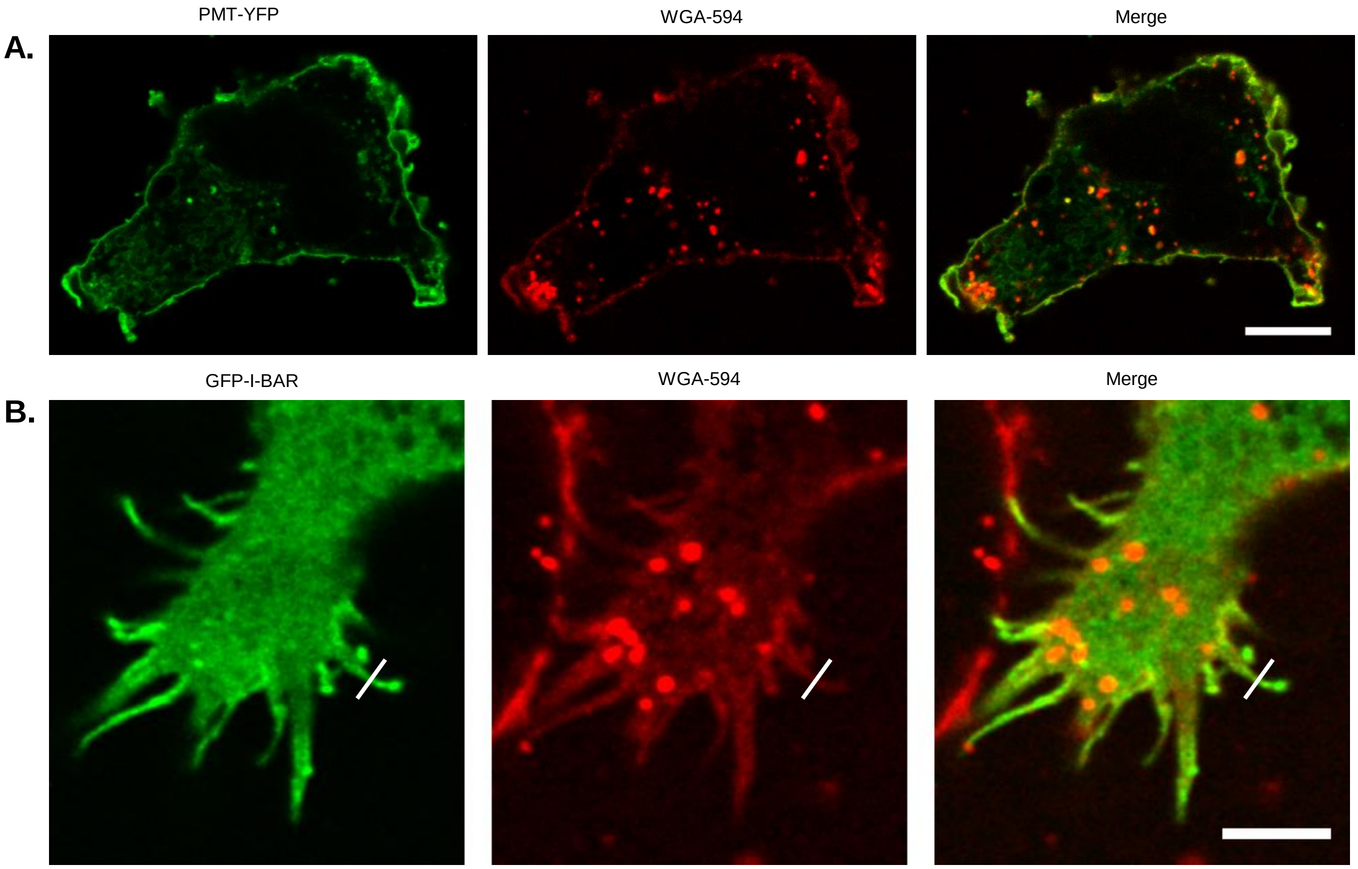

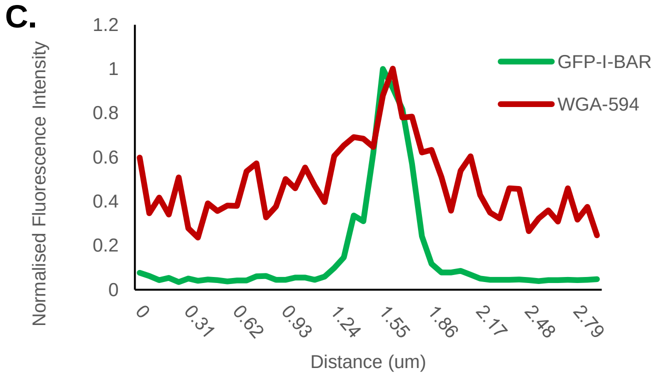

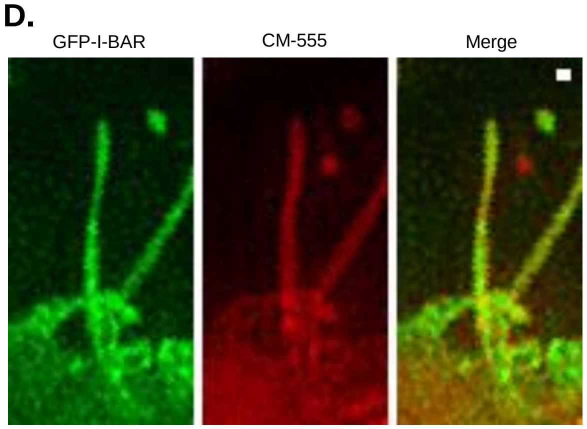

Supplementary Fig. S4.

(A). Comparison of membrane specific label plasma membrane targeted YFP (PMT-YFP) and plasma membrane specific staining dye wheat germ agglutinin (WGA-594). Representative confocal image showing N1E-115 cells expressing PMT-YFP was further stained for plasma membrane using membrane specific dye WGA-594 (scale bar = 10 µm). (B) Membrane specific dye label co-localisation within filopodia. Representative confocal image showing N1E-115 cells expressing GFP-I-BAR filopodia was stained with WGA-594 dye for membrane localisation (scale bar = 5 µm). (C). Showing the intensity overlap plot of GFP-I-BAR expressing cell filopodia with WGA-594. The marked filopodia from figure (B) was used for the intensity overlap plot. (D). Structured illumination microscopy (3D-SIM) image showing N1E-115 cell filopodia expressing GFP-I-BAR stained with plasma membrane specific dye CellMask orange 555 (CM-555) for membrane localisation (scale bar = 1 µm).

Figure S5

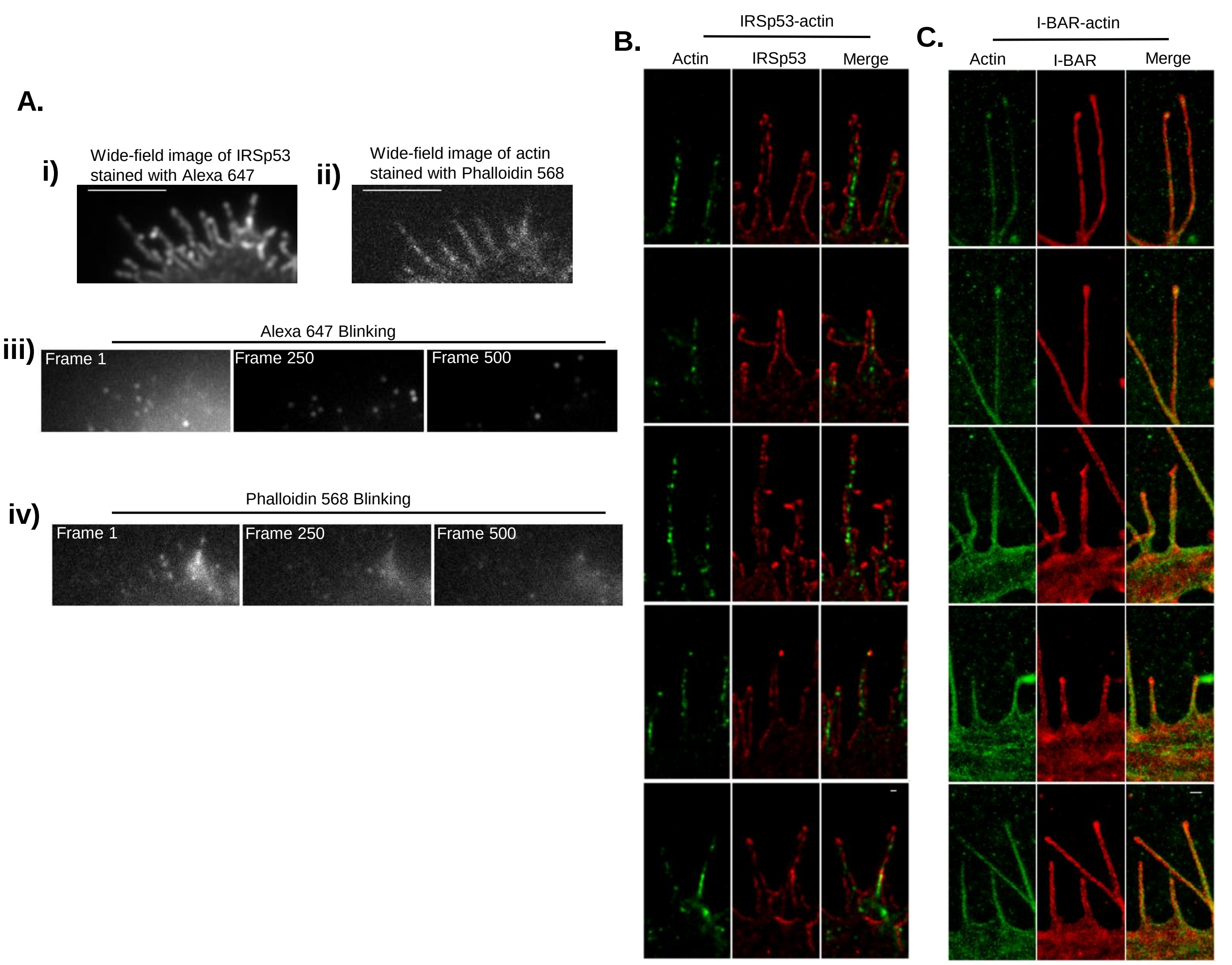

Supplementary Fig. S5.

(A) Shown is the representative ROI dual colour dSTORM image blinking pattern in N1E-115 cells (i) Wide-field image of HA-IRSp53 expressing cells stained with anti-HA Alexa 647 conjugate (ii) Wide-field image of endogenous actin stained with Alexa 568 Phalloidin. (iii). Blinking pattern of anti-HA Alexa 647 conjugate staining HA-IRSp53 at different frame points.(iv) Blinking pattern of endogenous actin stained with Alexa 568 at different frame points (scale bar = 5 µm).

(B) Montage of dual colour dSTORM images showing HA-IRSp53 expressing filopodia stained for –HA tag with anti-HA-Alexa 647 conjugate. Endogenous actin was stained with Alexa 568 Phalloidin (scale bar = 200 nm). (C) Montage of dual colour dSTORM images showing the GFP-I-BAR (domain of IRSp53) expressing filopodia stained for GFP with anti-GFP-Alexa 647 conjugate. Endogenous actin was stained with Alexa 568 Phalloidin (scale bar = 200 nm).

Figure S6

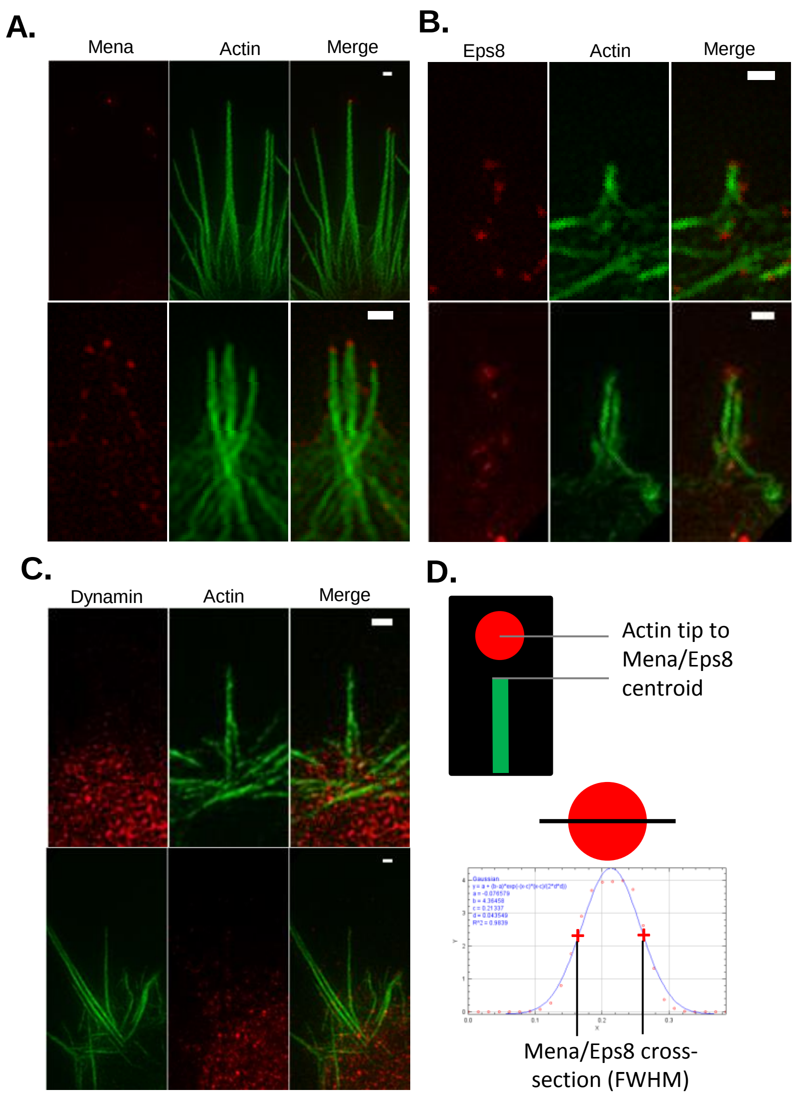

Supplementary Fig. S6.

Representative dual colour filopodia SIM images showing GFP fusions of IRSp53 (-SH3) domain interactors (Mena, Eps8 and Dynamin) along with HA-IRSp53 (not stained) and endogenous actin stained with Alexa 568 Phalloidin in N1E-115 cells. (A) GFP-Mena and endogenous actin (B) GFP-Eps8 and endogenous actin. (C) GFP-Dynamin and endogenous actin. For clarity purpose GFP fused (–SH3) interactors are shown red in colour and endogenous actin is shown in green in colour. Representative two filopodia images (top and bottom panel) are shown from each protein, for details on data processing see methods section (scale bar = 500 nm). (D) Methodology for determining actin tip to Mena/Eps8 centroid (manual measurements) and Mena/Eps8 cross-section FWHM (single Gaussian fitting and FWHM determination; (+ to +) measurements for Figs. 6D & E.

Figure S7

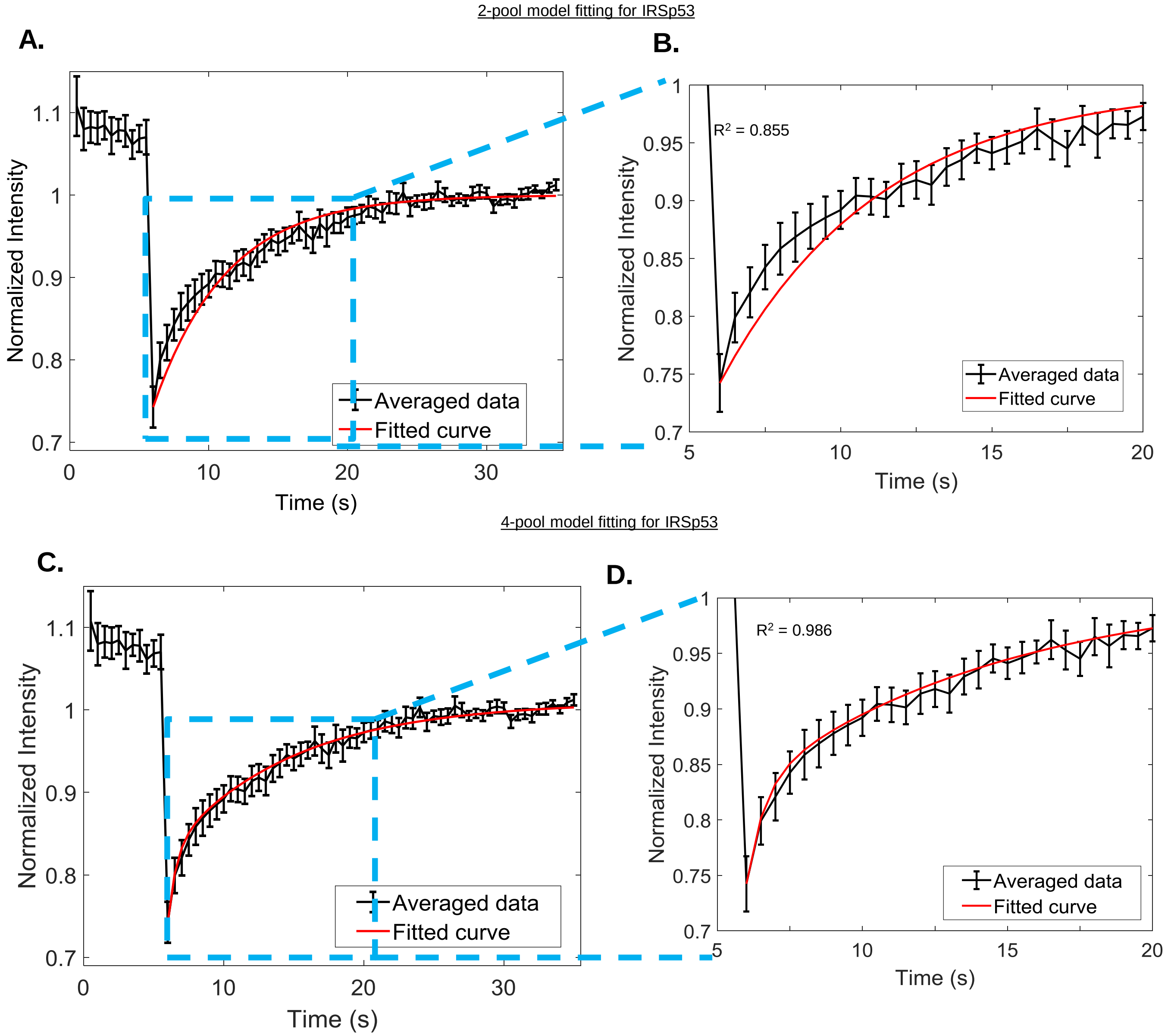

Supplementary Fig. S7.

Comparison of 2 & 4-pool model curve fitting.

(A) And (C). The FRAP recovery curve was calculated for each IRSp53 sample and then averaged across (n = 11) samples to produce the black curve. Error bars denote standard error. The red curve shows the fitted curve using maximum likelihood estimates from the 2-pool model (A) and 4-pool model (C). (B) and (D) show the region demarcated by blue box in (A) and (C) respectively. The R^2 value was obtained by comparing the FRAP recovery curve and the fitted curve between 6 to 16 seconds. From the FRAP recovery curves in (B) and (D), we can tell that the 2-pool model does not fit well and exhibit underfitting especially at the initial stages following photobleaching. Thus, the 4-pool model is more apt to explain the dynamics of IRSp53 protein.

Supplementary movie legends

Supplementary movie S1. The ROI movie of single molecule blinking pattern of frames shown in (fig S2 B) of anti-HA-Alexa 647 conjugate labelling on N1E-115 cells expressing HA-IRSp53.

Supplementary movie S2. Showing the representative ROI dual colour movie of frames shown in (fig S5 iii and iv). The blinking pattern of cells expressing HA-IRSp53 stained with anti-HA Alexa 647 conjugate and endogenous actin stained with Alexa 568 Phalloidin.
A). Movie is showing blinking pattern of anti-HA Alexa 647 conjugate staining HA-IRSp53.
B). Movie is showing blinking pattern of Alexa 568 staining endogenous actin.

Supplementary movie S3. Showing the representative ROI cross sectional view of three colour 3D SIM movie of filopodia expressing PMT-YFP and mRFP-I-BAR in N1E-115 cell. Cell was stained as described in methods with respective primary and secondary antibodies for each protein, briefly, PMT-YFP (Alexa 488), mRFP-I-BAR (Alexa 568) and endogenous actin with Alexa 647 Phalloidin. For clarity and contrast purpose the filopodia movie is shown as cyan (PMT-YFP), green (mRFP-I-BAR) and red (Phalloidin 647 for actin).

Supplementary movie S4. Showing the representative ROI cross sectional view of three colour 3D SIM movie of filopodia expressing PMT-YFP and mRFP-IRSp53 in N1E cell. The cell was stained as described in methods with respective primary and secondary antibodies for each protein, briefly, PMT-YFP (Alexa 488), mRFP-IRSp53 (Alexa 568) and endogenous actin with Alexa 647 Phalloidin. For clarity and contrast purpose the filopodia movie is shown as cyan (PMT-YFP), green (mRFP-IRSp53) and red (Phalloidin 647 for actin).

Supplementary movie S5. Time-lapse movie of a neurite in N1E-115 cell expressing mRFP-IRSp53 co-expressed with GFP-actin is shown (arrow indicates the dynamic filopodia gets shorten over time). The time-lapse movie was recorded every 30 s using wide-field fluorescence microscope. The filopodia dynamics was observed for a total of 12 min followed by the addition of Cytohalasin D (2 mM) at 7 min. The experiment was repeated three times and the representative movie is shown (scale bar = 5.0 µm).

Supplementary movie S6. Time-lapse movie of a N1E-115 cell expressing mRFP-I-BAR co-expressing GFP-actin is shown (arrow indicates the non-dynamic filopodia). The time-lapse movie was recorded every 30 s using wide-field fluorescence microscope. The filopodia dynamics was observed for a total of 12 min followed by the addition of Cytochalasin D (2 mM) at 7 min. The experiment was repeated three times and the representative movie is shown (scale bar = 5.0 µm).
